# Gα/GSA-1 works upstream of PKA/KIN-1 to regulate calcium signaling and contractility in the *Caenorhabditis elegans* spermatheca

**DOI:** 10.1371/journal.pgen.1008644

**Published:** 2020-08-10

**Authors:** Perla G. Castaneda, Alyssa D. Cecchetelli, Hannah N. Pettit, Erin J. Cram

**Affiliations:** Department of Biology, Northeastern University, Boston, MA, United States; University of Vermont College of Medicine, UNITED STATES

## Abstract

Correct regulation of cell contractility is critical for the function of many biological systems. The reproductive system of the hermaphroditic nematode *C*. *elegans* contains a contractile tube of myoepithelial cells known as the spermatheca, which stores sperm and is the site of oocyte fertilization. Regulated contraction of the spermatheca pushes the embryo into the uterus. Cell contractility in the spermatheca is dependent on actin and myosin and is regulated, in part, by Ca^2+^ signaling through the phospholipase PLC-1, which mediates Ca^2+^ release from the endoplasmic reticulum. Here, we describe a novel role for GSA-1/Gα_s,_ and protein kinase A, composed of the catalytic subunit KIN-1/PKA-C and the regulatory subunit KIN-2/PKA-R, in the regulation of Ca^2+^ release and contractility in the *C*. *elegans* spermatheca. Without GSA-1/Gα_s_ or KIN-1/PKA-C, Ca^2+^ is not released, and oocytes become trapped in the spermatheca. Conversely, when PKA is activated through either a gain of function allele in GSA-1 (GSA-1(GF)) or by depletion of KIN-2/PKA-R, the transit times and total numbers, although not frequencies, of Ca^2+^ pulses are increased, and Ca^2+^ propagates across the spermatheca even in the absence of oocyte entry. In the spermathecal-uterine valve, loss of GSA-1/Gα_s_ or KIN-1/PKA-C results in sustained, high levels of Ca^2+^ and a loss of coordination between the spermathecal bag and sp-ut valve. Additionally, we show that depleting phosphodiesterase PDE-6 levels alters contractility and Ca^2+^ dynamics in the spermatheca, and that the GPB-1 and GPB-2 G_β_ subunits play a central role in regulating spermathecal contractility and Ca^2+^ signaling. This work identifies a signaling network in which Ca^2+^ and cAMP pathways work together to coordinate spermathecal contractions for successful ovulations.

## Author summary

Organisms are full of biological tubes that transport substances such as food, liquids, and air through the body. Moving these substances in a coordinated manner, with the correct directionality, timing, and rate is critical for organism health. In this study we used *Caenorhabditis elegans*, a small transparent worm, to study how cells in biological tubes coordinate how and when they squeeze and relax. The *C*. *elegans* spermatheca is part of the reproductive system, which uses calcium signaling to drive the coordinated contractions that push fertilized eggs out into the uterus. Using genetic analysis and a calcium-sensitive fluorescent protein, we show that the G-protein GSA-1 functions with protein kinase A to regulate calcium release, and contraction of the spermatheca. These findings establish a link between G-protein and cAMP signaling that may apply to similar signaling pathways in other systems.

## Introduction

Regulation of cellular contractility and relaxation is essential for the function of epithelial and endothelial tubes, which are subjected to changing flow, strain, and pressure as they transport liquid, gases, and other cells throughout the body [1,2]. The *Caenorhabditis elegans* spermatheca, part of the hermaphroditic reproductive system, is an excellent model for the study of coordinated cell contractility [3–8]. The hermaphrodite reproductive system is composed of two symmetrical gonad arms surrounded by smooth muscle-like sheath cells, the spermathecae, and a common uterus [9,10]. The spermatheca, the site of fertilization, consists of an 8-cell distal neck, a 16-cell central bag, and a syncytial 4-cell spermatheca-uterine valve (sp-ut valve). During ovulation, gonadal sheath cells contract and the distal neck of the spermatheca is pulled open to allow entry of the oocyte. After a regulated period of time the sp-ut valve opens and the distal spermathecal bag constricts to expel the embryo, [11,12]. Failure to coordinate these distinct contraction events during ovulation leads to embryos that fail to reach the uterus, become misshapen, or have decreased viability [4,7].

The signaling pathways that regulate acto-myosin contractility in the spermatheca are similar to those found in smooth muscle and non-muscle cells [13–15]. Two pathways, a Ca^2+^ dependent and a Rho-dependent pathway are both necessary for spermathecal contractility. These pathways culminate in the phosphorylation and activation of non-muscle myosin. The Ca^2+^ dependent pathway relies on the activation of PLC-1/phospholipase C-ε, which cleaves phosphatidyl inositol (PIP_2_) into the second messengers diacylglycerol (DAG) and inositol 1,4,5 triphosphate (IP_3_). IP_3_ stimulates the release of Ca^2+^ from the endoplasmic reticulum (ER) through the ITR-1/IP_3_ receptor [3,5]. Cytosolic Ca^2+^ activates the MLCK-1/myosin light chain kinase, which phosphorylates and activates myosin [16]. The Rho-dependent pathway is activated by oocyte entry, which displaces a mechanosensitive Rho GAP, SPV-1, from the membrane, leading to the activation of RHO-1 [7]. GTP-bound RHO-1 then activates LET-502/ROCK, which in turn phosphorylates myosin and inhibits the myosin phosphatase, subsequently increasing the levels of phosphorylated myosin [5,7]. The sp-ut valve opens as the bag constricts to push the embryo into the uterus. Although Ca^2+^ signaling and actin-based contractility are critical [4,5,8,16–19], little is known about the mechanisms regulating the timing and spatial coordination of Ca^2+^ release during embryo transit through the spermatheca.

In order to better understand the regulation of Ca^2+^ signaling in the spermatheca, we performed a candidate RNA interference (RNAi) screen and identified the heterotrimeric G-protein alpha subunit GSA-1/Gα_s_ as an important regulator of spermathecal Ca^2+^ release. Gα_s_ is a GTPase that regulates diverse biochemical responses in multiple tissue types, including the contractility of airway and arteriole smooth muscle cells [20,21]. In *C*. *elegans*, GSA-1 regulates an array of behaviors including larval viability, egg laying, oocyte maturation and locomotion [22–25]. GSA-1 is expressed in neurons, muscle cells, pharynx and the male tail [25–27] and throughout the somatic gonad, including the sheath cells, the spermatheca, uterus and the vulval muscles [23,25], however, the role of GSA-1 in the spermatheca has not been studied.

Heterotrimeric G proteins are composed of α, β, and γ subunits and are typically found downstream of the 7-pass transmembrane class of G-protein coupled receptors (GPCR). Upon ligand binding, the GPCR acts as a guanine nucleotide exchange factor (GEF), exchanging GDP for GTP on the α subunit, and activating the heterotrimeric G protein. Heterotrimeric G proteins can also be activated via a receptor-independent mechanism facilitated by G protein regulator proteins (GPRs) [20,28]. Upon activation, the GTP-bound α subunit disassociates from the βγ subunit and both the α and βγ subunits can initiate downstream signaling pathways [29]. *C*. *elegans* express two Gβ subunits, encoded by GPB-1 and GPB-2. GPB-1 shares 86% homology with mammalian β subunits and interacts with all Gα subunits in *C*. *elegans* [30,31]. There are two γ subunits, GPC-1 and GPC-2, in the *C*. *elegans* genome. GPC-1/γ is expressed in sensory neurons, while GPC-2/γ is expressed more broadly [32]. Gβγ subunits can regulate ion channels [33,34] including Ca^2+^ channels [35], as well as activate or inhibit adenylyl cyclase [36]. GPB-2 acts downstream of the Gα subunit GOA-1 in pharyngeal pumping [37], and is required for egg-laying and locomotion [38,39].

In smooth muscle, activation of Gα_s_ inhibits acto-myosin contractility through the activation of adenylyl cyclase, which converts adenosine triphosphate (ATP) to 3’-5’-cyclic adenosine monophosphate (cAMP). cAMP levels are in part regulated by phosphodiesterases (PDEs), which convert cAMP into AMP. PKA is composed of two catalytic (PKA-C) and two regulatory (PKA-R) subunits, and is activated when cAMP binds to the PKA-R subunits, causing the release of PKA-C. Therefore, PKA-R acts as an inhibitor of PKA-C in the absence of cAMP. Once released, PKA-C interacts with multiple downstream effectors and regulates lipid metabolism [40], cell migration [41], and vasodilation [42], among many other functions [43]. In airway smooth muscle, PKA inhibits contraction by antagonizing Gα_q_ mediated increases in Ca^2+^, as well as by phosphorylating and inhibiting Rho [44] and other regulators of contractility. In *C*. *elegans*, PKA is encoded by catalytic subunit *kin-1/*PKA-C and regulatory subunit *kin-2/*PKA-R. KIN-1 has been implicated in a variety of functions, such as cold tolerance [45], oocyte meiotic maturation [46], locomotion [47], immune responses [48], and Ca^2+^ influx into motor neurons [49].

Here we describe a role for GSA-1/Gα_s_ and its downstream effectors, KIN-1/PKA-C and KIN-2/PKA-R, in the regulation of Ca^2+^ release and contractility in the *C*. *elegans* spermatheca. Dysregulation of these proteins results in abnormal Ca^2+^ signaling and loss of coordinated contractility between the sp-ut valve and the spermathecal bag. Additionally, we describe a role for Gβ subunits GPB-1 and GPB-2 and phosphodiesterase PDE-6 in spermathecal contractility and Ca^2+^ signaling.

## Results

### GSA-1/Gα_s_ and KIN-1/PKA-C are necessary for proper oocyte transit through the spermatheca

**Fig 1:**
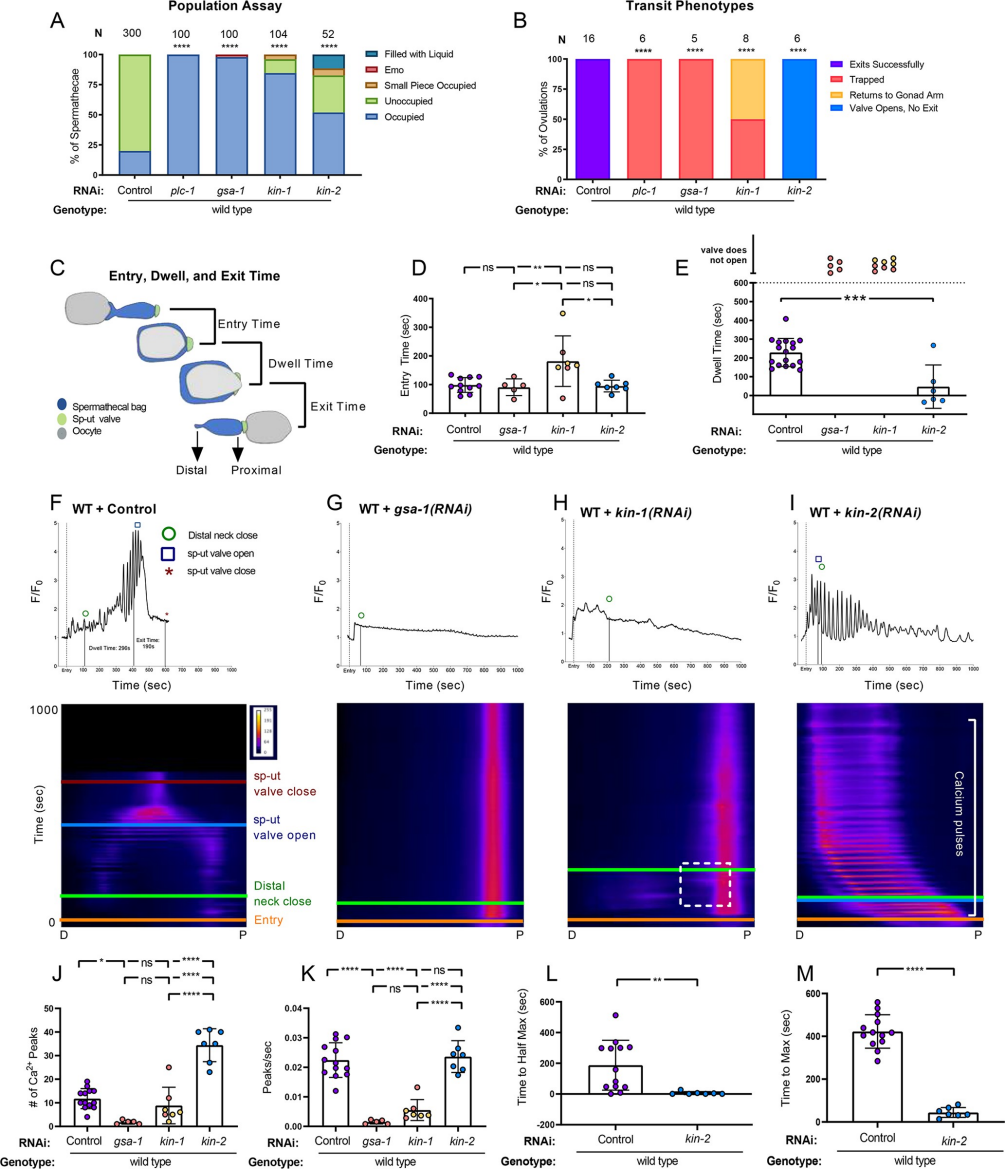
GSA-1/Gαs and KIN-1/PKA-C, and KIN-2/PKA-R regulate contractility and Ca^2+^ dynamics in the spermatheca. (A) Population assay of wild type animals grown on control RNAi, *plc-1(RNAi)*, *gsa-1(RNAi)*, *kin-1(RNAi)*, and *kin-2(RNAi)*. Spermathecae were scored for the presence or absence of an embryo (occupied or unoccupied), presence of a fragment of an embryo or presence of liquid (small piece occupied, filled with liquid), or the presence of endomitotic oocytes in the gonad arm (emo). The total number of unoccupied spermatheca were compared to the sum of all other phenotypes using the Fisher’s exact *t*-test. N is the total number of spermathecae counted. (B) Transit phenotypes of the ovulations of wild type animals treated with control RNAi, *plc-1(RNAi)*, *gsa-1(RNAi)*, *kin-1(RNAi)*, and *kin-2(RNAi)* and scored for successful embryo transits through the spermatheca (exits successfully), failure to exit (trapped), reflux into the gonad arm (returns to gonad arm), and the situation in which the sp-ut valve opens, but the embryo does not exit (valve opens, no exit). For transit phenotype analysis, statistics were performed using the total number of oocytes that exited the spermatheca successfully compared to the sum of all other phenotypes. Fisher’s exact *t*-tests were used for both population assays and transit phenotype analysis, and control RNAi was compared between all other RNAi treatments. (C) Schematic representation of a spermatheca undergoing an ovulation, with entry, dwell, and exit times indicated. Entry time (D) and dwell time (E) analysis of animals treated with control, *gsa-1(RNAi)*, *kin-1(RNAi)*, and *kin-2(RNAi)*. Color coding of the data points corresponds to the transit phenotypes in B. One-way ANOVA with a multiple comparison Tukey’s test was conducted to compare dwell times, and Fishers exact *t*-test (two dimensional *x*^*2*^ analysis) was performed on the exit times. Representative normalized Ca^2+^ traces and kymograms of movies in B (F-I) are shown with time of entry, distal neck closure, and time the sp-ut valve opens and closes indicated. Levels of Ca^2+^ signal were normalized to 30 frames before oocyte entry. Kymograms generated by averaging over the columns of each movie frame (see methods). Refer to S3A–S3D Fig for additional Ca^2+^ traces and S4A–S4D Fig for additional kymograms. Refer to S3H Fig for Ca^2+^ traces of wild type animals treated with *plc-1(RNAi)*. (J-M) Color coding of the data points corresponds to the transit phenotypes in B. (J) The number of Ca^2+^ peaks and (K) the peaks per second was determined for animals treated with control, *gsa-1(RNAi)*, *kin-1(RNAi)*, and *kin-2(RNAi)*, and were compared using one-way ANOVA with a multiple comparison Tukey’s test. The amount of time after oocyte entry required to reach either the (L) half maximum or (M) maximum Ca^2+^ signal was quantified for animals treated with control and *kin-2(RNAi)* and compared using Fishers exact *t*-test. Stars designate statistical significance (**** p<0.0001, *** p<0.005, ** p<0.01, * p<0.05).

To determine if GSA-1/Gα_s_ is required for oocyte transit, we depleted it using RNA interference (RNAi) in animals expressing GCaMP3 (GFP-calmodulin-M13 Peptide, version 3), a genetically encoded Ca^2+^ sensor, in the spermatheca [5,50]. Expression of GCaMP3 in the spermatheca provided both a visible GFP outline for scoring and a sensor for Ca^2+^ levels. The percentage of spermathecae occupied by an embryo was scored in adult animals. Depletion of factors that play a role in embryo transit through the spermatheca results in an increase in the percentage of occupied spermathecae compared to empty vector, or control RNAi, treated animals (18% occupied, n = 300). We used *plc-1*/PLC-ε, a gene essential for embryo transit, as a positive control (100% occupied, n = 214) [3–5] (Fig 1A).

Depletion of *gsa-1* by RNAi in GCaMP3 expressing animals resulted in almost complete spermathecal occupancy (98% occupied, n = 61) with a low penetrance entry defect, in which gonad arms were filled with endomitotically duplicating oocytes (EMO) [51] (2% EMO, n = 61;Fig 1A). The EMO phenotype occurs when mature oocytes fail to enter the spermatheca and re-enter the mitotic cycle, accumulating DNA [51]. Depletion of GSA-1 resulted in 90% spermathecal occupancy (Fig 1B), with the other 10% exhibiting entry defects (n = 87). The null allele *gsa-1(pk75)* results in larval arrest, and therefore cannot be used to study the role of GSA-1 in the spermatheca [24]. These results reveal a novel role for GSA-1 in the regulation of spermathecal contractility.

Because GSA-1/Gα_s_ often stimulates the production of cAMP and activation of PKA, we next explored a possible role for PKA in regulation of spermathecal contractility. KIN-1/PKA-C is expressed in the spermathecal bag and the sp-ut valve [40]. Depletion of KIN-1/PKA-C resulted in significantly increased spermathecal occupancy (85% occupied, n = 104; Fig 1A). Depletion of the regulatory subunit KIN-2/PKA-R also resulted in increased spermathecal occupancy (52% occupied, n = 52; Fig 1A,). These results suggest that regulation of PKA activity is critical for successful transit of fertilized embryos through the spermatheca.

### GSA-1/Gα_s_ KIN-1/PKA-C, and KIN-2/PKA-R regulate spermathecal contractility and Ca^2+^ dynamics

To better understand how GSA-1/Gα_s_, KIN-1/PKA-C, and KIN-2/PKA-R regulate transit of oocytes through the spermatheca, we scored time-lapse movies of animals expressing GCaMP3 in the spermatheca for the presence of four transit phenotypes: successful exit of the embryo (Exited Successfully), retention of the embryo in the spermatheca (Trapped), reflux of the embryo into the oviduct (Returned to Gonad Arm), and failure of the embryo to exit, despite an open sp-ut valve (Valve Opens, No Exit). As expected, all control RNAi embryos exited the spermatheca (100% Exited Successfully, n = 16; Fig 1B,) and all *gsa-1(RNAi)* embryos failed to exit the spermatheca (100% Trapped, n = 5). Unlike wild type ovulations (S1 Movie), in *gsa-1(RNAi)* animals, the embryo remained in the spermatheca with the distal neck and sp-ut valve fully closed (S2 Movie). In *kin-1(RNAi)* animals, 50% of the embryos remained in the spermatheca, while the others returned to the gonad arm (n = 8). We observed embryos that entered, returned to the gonad arm, and re-entered the spermatheca multiple times during imaging, while the sp-ut valve remained closed (S3 Movie). In 100% of the *kin-2(RNAi)* ovulations, the sp-ut valve opened, often prematurely, but the spermatheca failed to produce the contractions necessary for the embryo to be pushed into the uterus (n = 6).

To further quantify the transit dynamics, we measured the entry time, dwell time, and exit time [16,52] in the time lapse movies. The entry time is the time from the beginning of oocyte entry to when the distal neck closes, fully enclosing the oocyte in the spermatheca. The dwell time is measured from time of distal neck closure to the point the sp-ut valve begins to open. The exit time is measured from sp-ut valve opening, through expulsion of the fertilized embryo into the uterus and full closure of the sp-ut valve (Fig 1C). Entry times in *gsa-1(RNAi)* and *kin-2(RNAi)* did not differ significantly from wild type. However, because of the defects in distal neck closure discussed above, *kin-1(RNAi)* animals had a significantly increased entry time (Fig 1D). Because the sp-ut valve never opens in *gsa-1(RNAi)* and *kin-1(RNAi)* animals, neither dwell time nor exit time could be measured. This is reflected by the color-coded values annotated “valve does not open” in Fig 1D. In contrast, in *kin-2(RNAi)* animals, the sp-ut valve opened significantly faster than wild type, resulting in low, and sometimes negative, dwell times (Fig 1E). Despite these short dwell times, the oocytes consistently failed to exit the spermatheca during the 30-minute imaging period, resulting in no exit times for *kin-2(RNAi)* animals.

Taken together, these results are consistent with the hypothesis that PKA negatively regulates contractility in the spermatheca and sp-ut valve. We propose that when KIN-1/PKA-C is depleted, increased contractility in the proximal bag and sp-ut valve prevent exit of the fertilized embryo. In this case, the embryo is trapped or pushed back into the oviduct. Conversely, when KIN-2/PKA-R is depleted, activating KIN-1/PKA-C, neither the spermathecal bag nor sp-ut valve is contractile, resulting in reduced exit of the fertilized embryos. In this case, the embryo is not pushed out.

Because Ca^2+^ signaling plays a key role in spermatheca contractility [5], we next explored the role of GSA-1 and PKA in spermathecal Ca^2+^ dynamics. We collected time-lapse fluorescent images of animals expressing GCaMP3 and plotted the data both as 1D traces of total Ca^2+^ levels, and as 2D kymographs with time on the y-axis and space in the x-axis, which allows visualization of the spatial and temporal aspects of Ca^2+^ signaling. In wild type animals, the sp-ut valve exhibits a bright pulse of Ca^2+^ immediately upon oocyte entry, which is followed by a quiet period. Once the oocyte is completely enclosed, Ca^2+^ oscillates across the spermatheca, increasing in intensity until peaking concomitantly with distal spermathecal constriction and embryo exit [5,52] (Fig 1F, S1 Movie). Depletion of *gsa-1(RNAi)* resulted in low levels of Ca^2+^ signal in the spermathecal bag. In contrast, Ca^2+^ signal in the sp-ut valve remained high throughout the entire observation period (n = 5; Fig 1G; S2 Movie). We observed similarly low levels of Ca^2+^ signal in *kin-1(RNAi)* animals, however, some Ca^2+^ signal was observed on the proximal side of the spermathecal bag (n = 8; Fig 1H, indicated by box; S2 Movie). This increased proximal Ca^2+^ signal (Fig 1H) coincided with retrograde motion of the embryo back into the gonad arm (see Fig 1B; S3 Movie). These results suggest GSA-1 and KIN-1 are required for proper levels of Ca^2+^ signaling in the spermathecal bag and for the correct inhibition of Ca^2+^ in the sp-ut valve.

We next investigated the effect of hyperactivation of the catalytic subunit KIN-1/PKA-C by depleting the regulatory subunit, KIN-2/PKA-R. Because depletion of KIN-1 results in a loss of Ca^2+^ signaling in the spermatheca bag, we expected that the loss of the regulatory subunit, KIN-2/PKA-R, might increase Ca^2+^ signaling by allowing the catalytic subunit to remain uninhibited. In *kin-2(RNAi)* animals, Ca^2+^ signaling increased immediately upon oocyte entry (Fig 1I). Ca^2+^ transients propagated from the distal to the proximal side of the spermatheca in rhythmic succession (S4 Movie, S1 Fig). These Ca^2+^ transients appeared as pulses in the Ca^2+^ trace data and as horizontal lines in the kymogram (Fig 1I). Although *kin-2(RNAi)* stimulated Ca^2+^ release, embryos failed to exit the spermatheca (n = 7, Fig 1B). These results suggest unregulated activity of KIN-1/PKA-C, through loss of KIN-2/PKA-R, results in abnormal Ca^2+^ signaling in the spermatheca, which is insufficient to stimulate embryo exit.

To quantify the differences in Ca^2+^ signal and enable statistical analysis over many embryo transits, we used Matlab scripts to identify peaks in the GCaMP time series. To capture the rate of increase in calcium signal during embryo transit, we quantified the amount of time after oocyte entry required to reach half the maximum Ca^2+^ signal [52]. To determine the length of time required for the strongest Ca^2+^ pulses to occur, we calculated the time to the maximum peak value. To quantitatively define ‘flat’ traces, we calculated the variance of the first derivative, which expresses how quickly the intensity values in the data series are changing with respect to time [53]. As described above, when signal through GSA-1 or PKA was reduced by *gsa-1(RNAi)* and *kin-1(RNAi)*, few transients were seen and the Ca^2+^ traces were significantly flatter than the wild type control (Compare Fig 1F to Fig 1G and 1H; S2A Fig). Because of these flat profiles, both *gsa-1(RNAi)* and *kin-1(RNAi)* exhibited significantly reduced Ca^2+^ peaks (Fig 1J), and Ca^2+^ peaks per second (Fig IK). Because the *gsa-1(RNAi)* and *kin-1(RNAi)* traces did not increase in intensity, the time to half max (Fig 1L) and time to max metrics are not biologically informative metrics. In contrast, when PKA activity was increased through the depletion of KIN-2/PKA-R, the number of Ca^2+^ peaks increased significantly (Fig 1J), however, when we accounted for the duration of the transit, peak frequency per se was not significantly different than wild type (Fig 1K). In *kin-2(RNAi)* animals, onset of Ca^2+^ transients occurred almost immediately after oocyte entry, resulting in a significantly reduced time to both half maximum (Fig 1L) and maximum Ca^2+^ signal (Fig 1M). These results support the conclusion that GSA-1 and KIN-1/PKA stimulate the production of Ca^2+^ transients in the *C*. *elegans* spermatheca.

### GSA-1(GF) induction of Ca^2+^ signaling in the spermatheca is dependent on KIN-1/PKA-C and PLC-1

**Fig 2:**
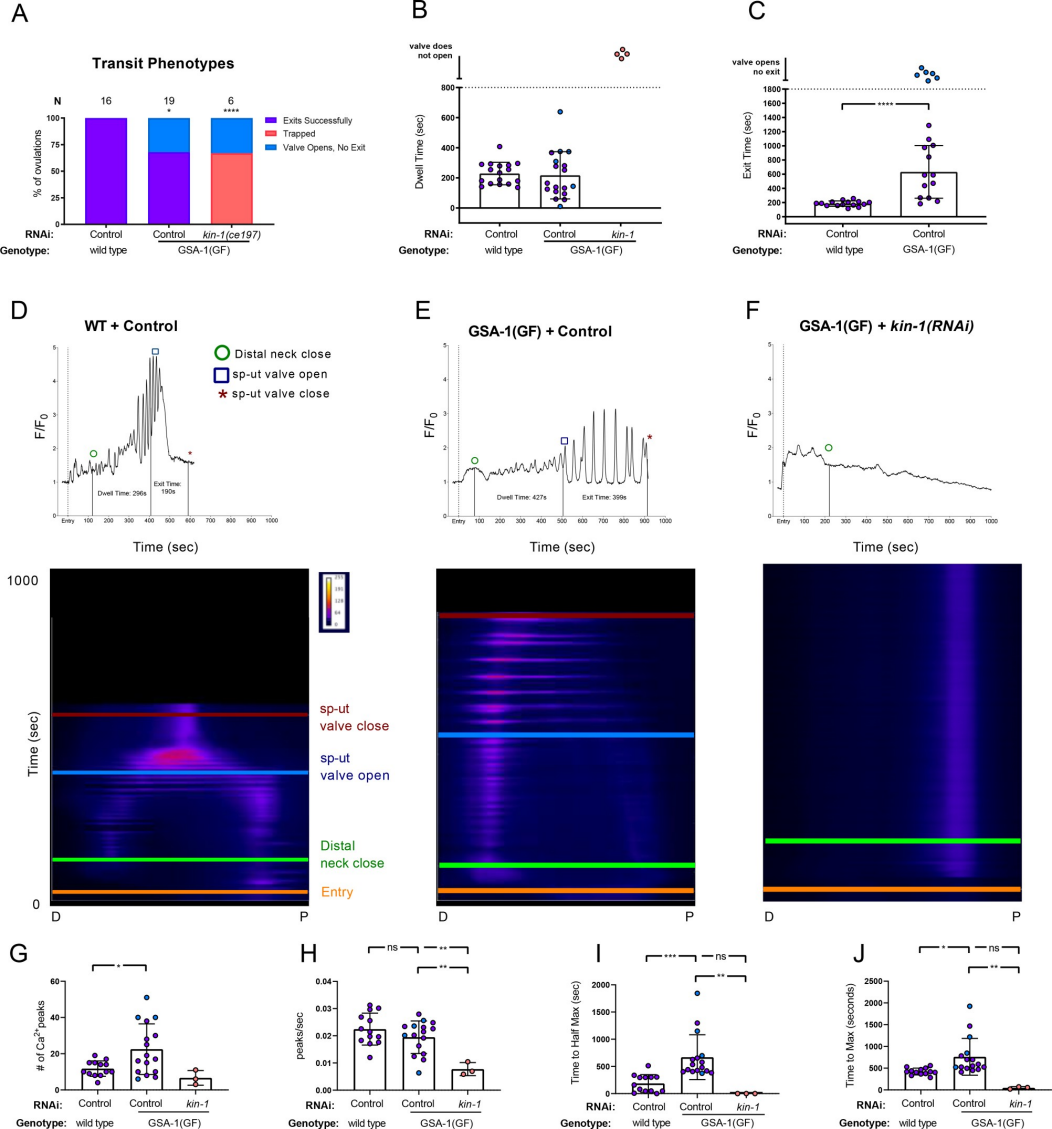
GSA-1(GF) induction of Ca^2+^ signaling in the spermatheca is dependent on KIN-1/PKA-C. (A) Transit phenotypes of the ovulations of wild type animals treated with control RNAi, and GSA-1(GF) animals treated with control RNAi and *kin-1(RNAi)*, were scored for successful embryo transits through the spermatheca (exits successfully), failure to exit (trapped), reflux into the gonad arm (returns to gonad arm), and the situation in which the sp-ut valve opens, but the embryo does not exit (valve opens, no exit). The total number of oocytes that exited the spermatheca successfully was compared to the sum of all other phenotypes using the Fisher’s exact *t*-test. Control RNAi was compared to all other RNAi treatments. Dwell (B) and exit (C) times of movies in A were analyzed using Fishers exact *t*-test (two dimensional *x*^*2*^ analysis). Color coding of the data points corresponds to the transit phenotypes in A. Representative normalized Ca^2+^ traces and kymograms of movies in A (D-F) are shown with time of entry, distal neck closure, and time the sp-ut valve opens and closes indicated. Levels of Ca^2+^ signal were normalized to 30 frames before oocyte entry. Kymograms generated by averaging over the columns of each movie frame (see methods). Refer to S3A Fig and S5A and S5B Fig for additional Ca^2+^ traces, and S4A Fig and S6A and S6B Fig for additional kymograms. (G-J) Color coding of the data points corresponds to the transit phenotypes in A. (G) The number of Ca^2+^ peaks, (H) the peaks per second, and the amount of time after oocyte entry required to reach either (I) half the maximum or (J) maximum Ca^2+^ signal were quantified for wild type animals treated with control RNAi, and GSA-1(GF) animals treated with control RNAi and *kin-1(RNAi)*. These were compared using one-way ANOVA with a multiple comparison Tukey’s test. Stars designate statistical significance (*** p<0.005, ** p<0.01, * p<0.05).

We next explored the effects of hyperactivating GSA-1 signaling on spermathecal transits. The allele *gsa-1(ce94)* is a gain of function allele (referred to here as GSA-1(GF)) in which the GTP-bound form of GSA-1 is stabilized, resulting in an elevated level of GSA-1 activity [54]. Most fertilized embryos in GSA-1(GF) animals passed successfully through the spermatheca, however, in 31% of transits, the sp-ut valve opened, but the embryo was unable to exit (n = 13; Fig 2A), suggesting that the bag may not contract with the timing or force needed to efficiently expel the embryo into the uterus (Fig 2C). We reasoned that if PKA is downstream of GSA-1, depletion of KIN-1/PKA-C in the GSA-1(GF) background would result in increased trapping of embryos in the spermatheca. Indeed, GSA-1(GF) animals treated with *kin-1(RNAi)* resulted in a complete failure of embryos to exit the spermatheca. We observed 67% of the embryos remained entirely enclosed by the spermatheca, while in the remaining 33% the valve opened but the embryo was unable to exit (n = 6; Fig 2A).

Because *gsa-1(RNAi)* reduced Ca^2+^ signaling in the spermathecal bag but increased signaling in the sp-ut valve, we predicted increasing GSA-1 activity would produce the opposite effect. However, rather than exhibiting prematurely elevated Ca^2+^, expression of GSA-1(GF) resulted in low amplitude peaks for the first few hundred seconds, after which Ca^2+^ was released in pulses that continued until embryo exit. In the sp-ut valve, Ca^2+^ signal was depressed, as expected (Compare Fig 2D and 2F). The number of Ca^2+^ peaks increased significantly in GSA-1(GF) animals (Fig 2G), however peak frequency was not significantly different than wild type (Fig 2H). This suggests that, as with depletion of KIN-2/PKA-R (see Fig 1) the observed increase in overall number of peaks in GSA-1(GF) is due to the increased transit lengths.

If PKA is downstream of GSA-1(GF), depletion of KIN-1/PKA-C should suppress the Ca^2+^ phenotypes seen in the GSA-1(GF) animals. Indeed, depletion of KIN-1 in GSA-1(GF) prevented the Ca^2+^ pulses observed in GSA-1(GF) animals (Fig 2G, S3B Fig), significantly decreasing the number of Ca^2+^ peaks (Fig 2H) and peaks per second (Fig 2I). Because the Ca^2+^ signal in *kin-1(RNAi)* animals did not ramp up over time, the highest amplitude peaks are reached early in the time series, which is reflected in the reduced time to half maximum (Fig 2I) and maximum Ca^2+^ signal (Fig 2J) in GSA-1(GF) animals treated with *kin-1(RNAi)*. These metrics are similar to those seen when wild type animals were treated with *kin-1(RNAi)* (See Fig 1G–1J). These results are consistent with the hypothesis that KIN-1 is acting downstream of GSA-1 in the spermatheca to regulate Ca^2+^ release.

**Fig 3:**
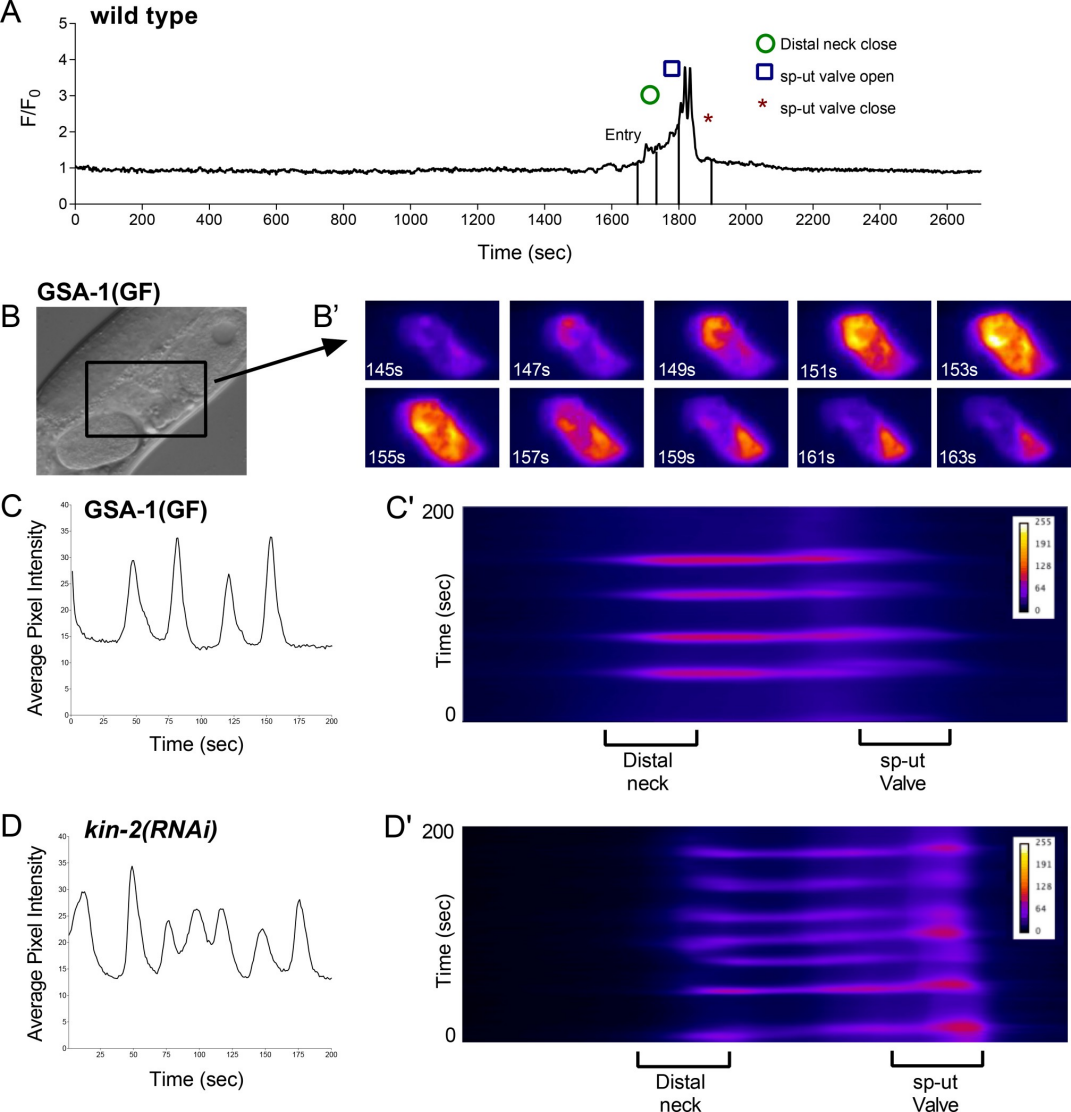
GSA-1(GF) and knockdown of KIN-2/PKA-R induce Ca^2+^ signaling in an empty spermatheca. (A) Normalized Ca^2+^ trace of a wild type ovulation before, during, and after transit. Ca^2+^ signal does not rise above basal levels before or after the transit. Time of entry, distal neck closure, and time the sp-ut valve opens and closes are indicated. (B) DIC image of a GSA-1(GF) animal; the spermatheca is indicated by a box. (B’) Ca^2+^ signaling in the unoccupied GSA-1(GF) spermatheca shown in B at an arbitrary timepoint before an ovulation event. Ca^2+^ repeatedly increases, peaks, and then drops to baseline levels. These pulses travel from the distal spermatheca through the bag to the sp-ut valve (S5 Movie). Average pixel intensity trace of the Ca^2+^ signal (C) and kymogram (C’) of the GSA-1(GF) spermatheca are shown. Knockdown of KIN-2 with RNAi results in a similarly pulsing unoccupied spermatheca, as shown by the average pixel intensity trace of the Ca^2+^ signal (D) and kymogram (D’) of a *kin-2(RNAi)* animal.

In wild type animals, oocyte entry stimulates Ca^2+^ release [5]. The spermathecal Ca^2+^ remains at baseline until ovulation and returns to baseline following embryo exit (Fig 3A). However, while observing GSA-1(GF) animals, we noticed Ca^2+^ pulses in unoccupied spermathecae, which traveled from the distal spermatheca through the bag to the sp-ut valve (Fig 3B and 3C, S5 Movie). Similarly, in *kin-2(RNAi)* animals, Ca^2+^ repeatedly increased, peaked, and then dropped to baseline levels in empty spermathecae (Fig 3D). These results suggest that activating GSA-1 or PKA can bypass the need for oocyte entry as a trigger for Ca^2+^ release. In addition, these similarities between GSA-1(GF) and *kin-2(RNAi)* support the hypothesis that GSA-1 triggers Ca^2+^ signaling through KIN-1/KIN-2. The GSA-1(GF) and *kin-2(RNAi)* Ca^2+^ traces share features, such as triggering Ca^2+^ signaling in empty spermathecae, and reducing the time to half max and max signal in occupied spermathecae (see Fig 1I and 1J and Fig 2I and 2J), however, activating PKA through *kin-2(RNAi)* produces high amplitude peaks sooner after oocyte entry. This difference could be a result of either the degree of activation of PKA, or differences in downstream effectors.

**Fig 4:**
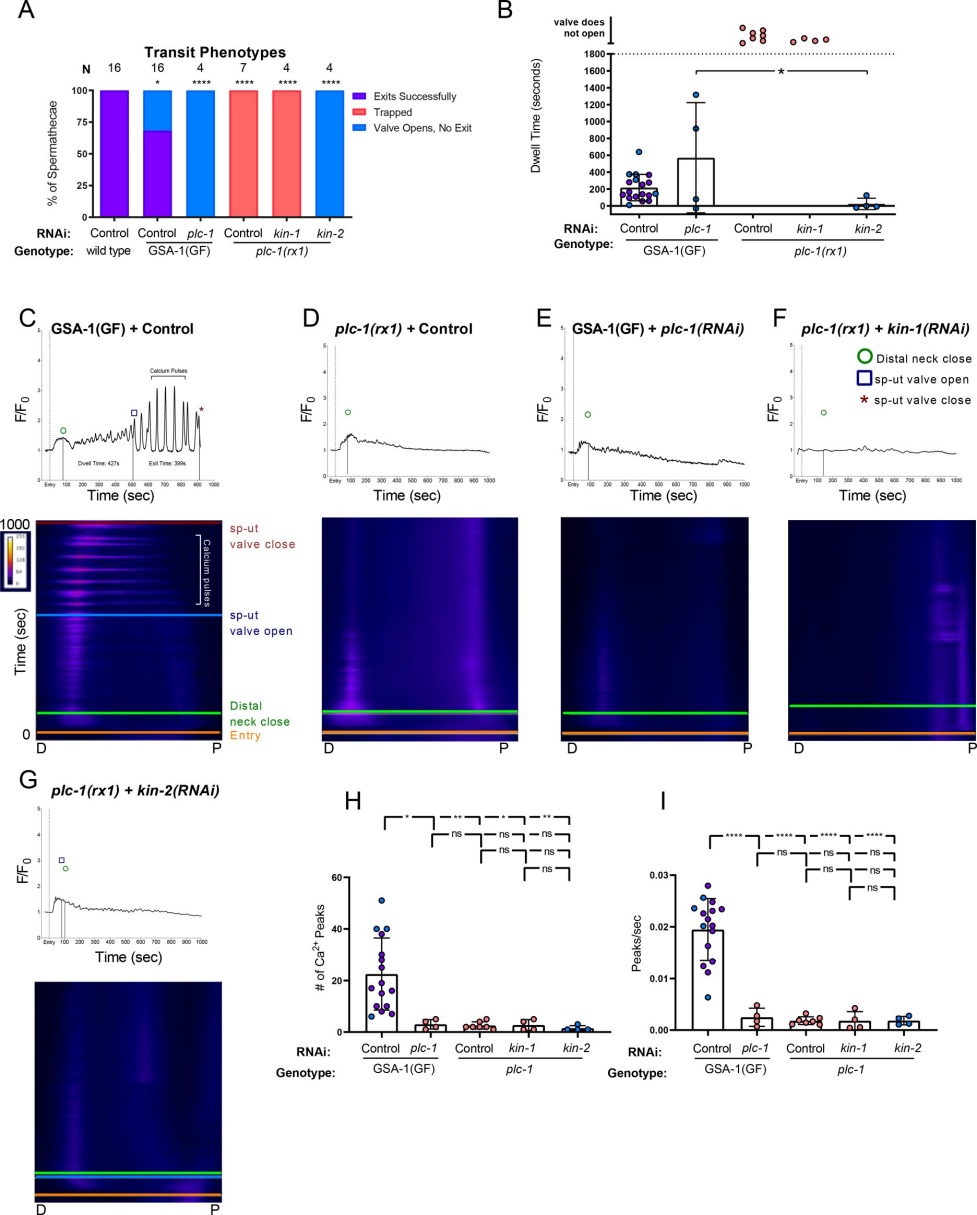
Ca^2+^ signal is dependent on PLC-1. (A) Transit phenotypes of the ovulations of wild type animals treated with control RNAi, GSA-1(GF) animals treated with control RNAi and *plc-1(RNAi)*, and *plc-1* null animals treated with control RNAi, *kin-1(RNAi)*, *and kin-2(RNAi)* were scored for successful embryo transits through the spermatheca (exits successfully), failure to exit (trapped), and the situation in which the sp-ut valve opens, but the embryo does not exit (valve opens, no exit). The total number of oocytes that exited the spermatheca successfully was compared to the sum of all other phenotypes using the Fisher’s exact *t*-test. Control RNAi was compared to all other RNAi treatments. (B) Dwell times derived from movies in A were compared using One-way ANOVA with a multiple comparison Tukey’s test. Color coding of the data points corresponds to the transit phenotypes in A. Representative normalized Ca^2+^ traces and kymograms of movies in A (C-G) are shown with time of entry, distal neck closure, and time the sp-ut valve opens and closes indicated. Levels of Ca^2+^ signal were normalized to 30 frames before oocyte entry. Kymograms generated by averaging over the columns of each movie frame (see methods). Refer to S5A and S5E Fig and S7A–S7C Fig for additional Ca^2+^ traces, and S5A Fig and S6A, S6C, and S8A–S8C Fig for additional kymograms. (H-I) Color coding of the data points corresponds to the transit phenotypes in A. (H) The number of Ca^2+^ peaks (see methods) and (I) the peaks per second was determined for GSA-1(GF) animals treated with control RNAi and *plc-1(RNAi)*, and *plc-1* null animals treated with control RNAi, *kin-1(RNAi)*, *and kin-2(RNAi)*. Both were compared using one-way ANOVA with a multiple comparison Tukey’s test. Stars designate statistical significance (**** p<0.0001, *** p<0.005, ** p<0.01, * p<0.05).

Previous work has shown that the phospholipase PLC-1 is required for spermathecal contractility and Ca^2+^ release [5]. Therefore, we next asked whether the phospholipase PLC-1 was required for transits in GSA-1(GF) animals. As expected, the null allele *plc-1(rx1)* resulted in 100% spermathecal occupancy (n = 7; Fig 4A). Similarly, in GSA-1(GF) animals treated with *plc-1(RNAi)*, embryos did not exit the spermatheca, however the sp-ut valve did open (n = 4; Fig 4A). Occasionally, the valve opened before the distal neck closed, resulting in negative dwell times (Fig 4B). We next asked if the Ca^2+^ signal observed in the GSA-1(GF) animals (Fig 4C) required phospholipase-stimulated Ca^2+^ release. In *plc-1* null animals, Ca^2+^ signaling remained at baseline levels in the spermathecal bag even after oocyte entry (Fig 6D). When GSA-1(GF) animals were treated with *plc-1(RNAi*), the Ca^2+^ signal remained low in the spermathecal bag, and we no longer observed the Ca^2+^ pulses characteristic of GSA-1(GF) (Fig 4E). Similarly, loss of *plc-1* prevented both the excess proximal Ca^2+^ release observed in *kin-1(RNAi)* spermathecae (Fig 4F), and the Ca^2+^ pulses observed in *kin-2(RNAi)* spermathecae (Fig 4G). In GSA-1(GF) and *kin-2(RNAi)* animals, the number of Ca^2+^ peaks (Fig 4H) and Ca^2+^ peaks per second (Fig 4I), were reduced to the level seen in *plc-1(RNAi*) animals. Because the intensity of the Ca^2+^ did not increase over time in these low variance traces (S2C Fig, S6E Fig, S8A–S8C Fig) the time to half max and time to max are not biologically informative metrics. These results suggest the Ca^2+^ pulses observed when either GSA-1 or PKA signaling is activated require PLC-1, and indicate GSA-1 and PKA act upstream or in parallel to PLC-1 to stimulate Ca^2+^ release in the spermatheca.

### Phosphodiesterase PDE-6 is necessary for normal transit times and Ca^2+^ dynamics

**Fig 5:**
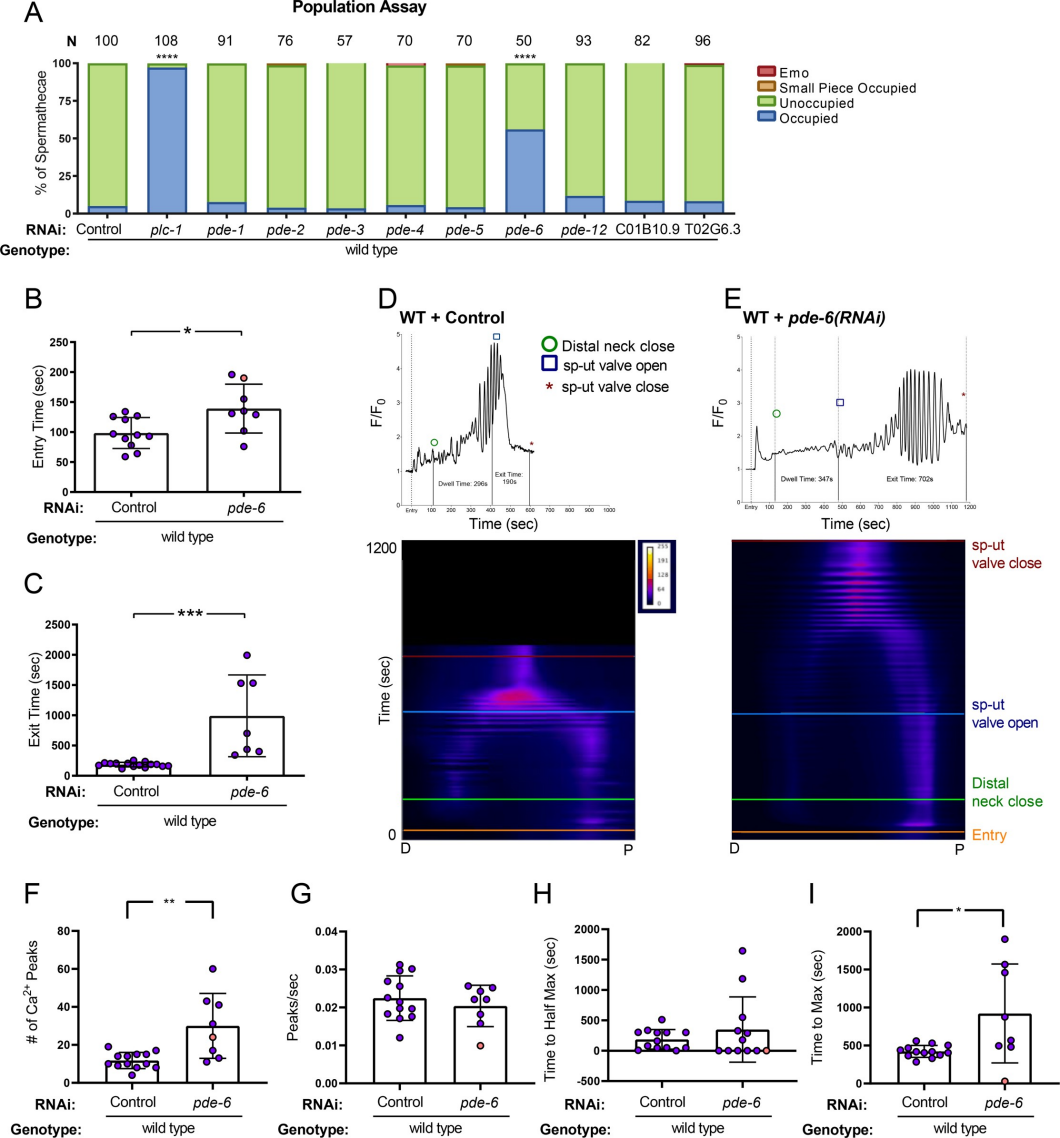
Phosphodiesterase PDE-6 is necessary for normal transit times and Ca^2+^ dynamics. (A) population assay of wild type animals grown on control RNAi, *plc-1(RNAi)*, *pde-1(RNAi)*, *pde-2(RNAi)*, *pde-3(RNAi)*, *pde-4(RNAi)*, *pde-5(RNAi)*, *pde-6(RNAi)*, *pde-12(RNAi)*, C01B10.9 and T02G6.3. Spermathecae were scored for the presence or absence of an embryo (occupied or unoccupied), presence of a fragment of an embryo (small piece occupied), or the presence of endomitotic oocytes in the gonad arm (emo) phenotypes. The total number of unoccupied spermatheca was compared to the sum of all other phenotypes using the Fisher’s exact *t*-test. N is the total number of spermathecae counted. Control RNAi was compared to all other RNAi treatments. Entry time (B) and exit time (C) were compared using Fisher’s exact *t*-test (two dimensional *x*^*2*^ analysis). (B,C) Values are color coded corresponding to transit phenotype (purple: successful exit, orange: traps. Representative normalized Ca^2+^ traces and kymograms of control RNAi (D) and *pde-6(RNAi)* (E) are shown with time of entry, distal neck closure, and time the sp-ut valve opens and closes indicated. Levels of Ca^2+^ signal were normalized to 30 frames before oocyte entry. Kymograms were generated by averaging over the columns of each movie frame (see methods). Refer to S3A Fig and S3G Fig for additional Ca^2+^ traces, and S4A Fig and S9A Fig for additional kymograms. (F-I) Values are color coded corresponding to transit phenotype (purple: successful exit, orange: traps). (F) The number of Ca^2+^ peaks, (G) the peaks per second, and the amount of time after oocyte entry required to reach either (H) half the maximum or (I) maximum Ca^2+^ signal were quantified for wild type animals treated with control and *pde-6(RNAi)*. Values were compared using Fisher’s exact *t*-test. Stars designate statistical significance (**** p<0.0001, *** p<0.005, ** p<0.01, * p<0.05).

Because PKA is activated by cAMP, we anticipated that altering cAMP levels in the spermatheca would alter the contractility and Ca^2+^ dynamics during ovulation. Phosphodiesterases (PDEs) are enzymes that regulate the concentration of cAMP by catalyzing its hydrolysis into AMP [55]. To determine whether increasing cAMP levels by depleting PDEs would affect spermathecal transits, we depleted each of the known *C*. *elegans* PDEs and identified PDE-6 as an important regulator of spermathecal contractility. Depletion of *pde-6* resulted in 56% (n = 50) of animals with embryos retained in the spermatheca (Fig 5A). To study the effects of PDE-6 on ovulations and Ca^2+^ dynamics, we collected time-lapse images of GCaMP3 animals fed *pde-6(RNAi)*. Both the entry time and the exit time in *pde-6(RNAi)* animals were significantly longer than wild type (Fig 5B and Fig 5C. In one case, the sp-ut valve never opened, and the embryo remained trapped in the spermatheca (Fig 5D). The *pde-6(RNAi)* ovulations resulted in delayed Ca^2+^ release in the bag, after which the Ca^2+^ was released in pulses until embryo exit (n = 8; Fig 5E). The increased number of peaks observed in *pde-6(RNAi*) (Fig 5F), correspond to the increased transit time, rather than an increase in overall peak frequency (Fig 5G). While the time to half maximum Ca^2+^ value is not significantly different from wild type (Fig 5H), the time to maximum is significantly increased (Fig 5I), due to the delay before the onset of high amplitude Ca^2+^ pulses. The *pde-6(RNAi)* Ca^2+^ metrics are similar to the GSA-1(GF) metrics (S10 Fig). These results suggest PDE-6 regulates Ca^2+^ signaling and spermathecal contractility, likely through regulation of cAMP levels.

### Heterotrimeric G-protein beta subunit GPB-1 and GPB-2 are required to regulate Ca^2+^ signaling and spermatheca contractility

**Fig 6:**
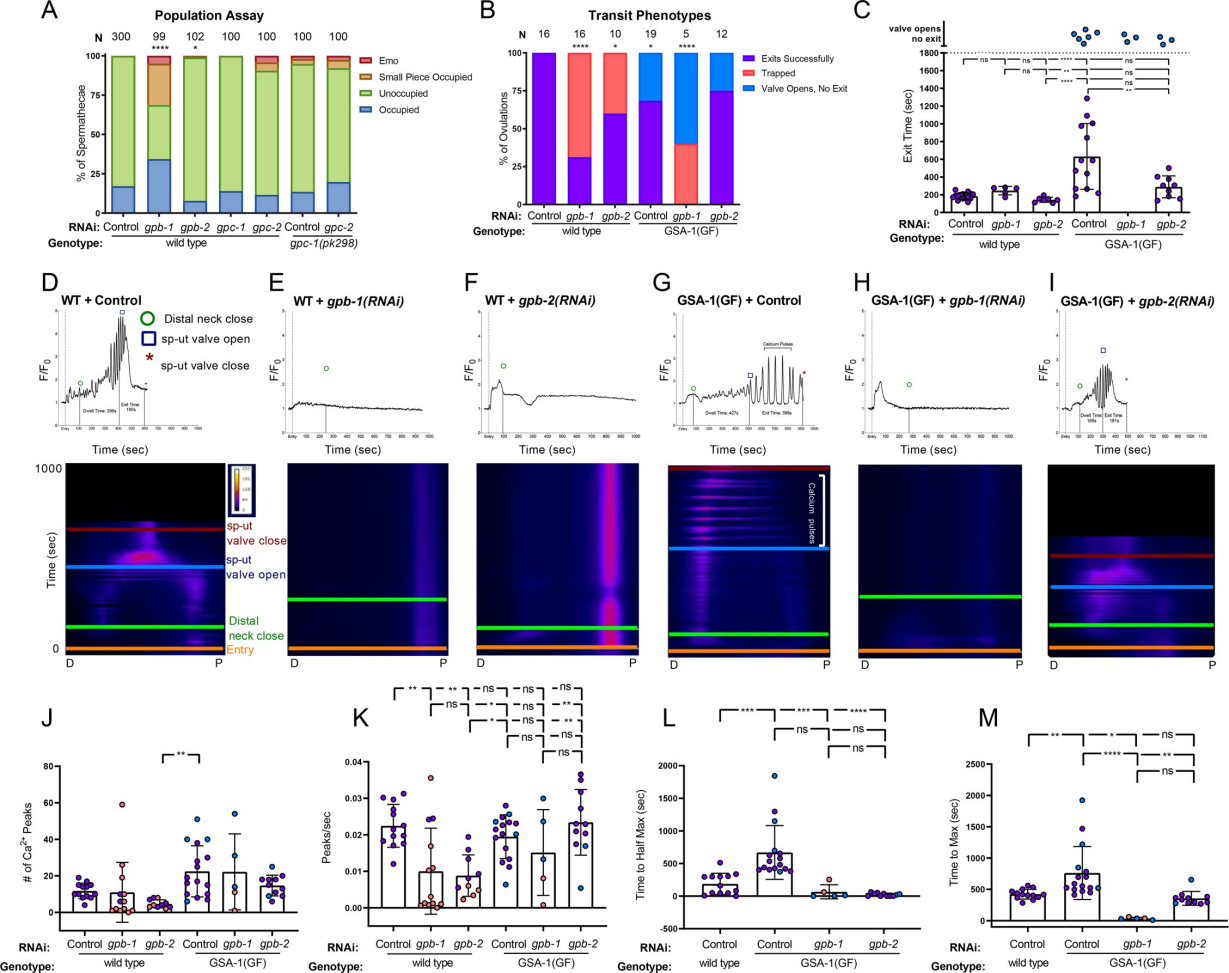
Heterotrimeric G-protein beta subunits GPB-1 and GPB-2 regulate Ca^2+^ signaling and spermatheca contractility. (A) Population assay of wild type young adults grown on control RNAi, *gpb-1(RNAi)*, *gpb-2(RNAi)*, *gpc-1(RNAi)*, and *gpc-2(RNAi)* and *gpc-1(pk298)* grown on control RNAi and *gpc-2(RNAi)*. Spermathecae were scored for the presence or absence of an embryo (occupied or unoccupied), presence of a fragment of an embryo (small piece occupied), or the presence of endomitotic oocytes in the gonad arm (emo) phenotypes. The total number of unoccupied spermatheca was compared to the sum of all other phenotypes using the Fisher’s exact *t*-test. N is the total number of spermathecae counted. (B) Transit phenotypes of the ovulations in wild type animals treated with control RNAi, *gpb-1(RNAi)* and *gpb-2(RNAi)*, and GSA-1(GF) animals treated with control RNAi, *gpb-1(RNAi)*, and *gpb-2(RNAi)* were scored for successful embryo transits through the spermatheca (exits successfully), failure to exit (trapped), and the situation in which the sp-ut valve opens, but the embryo does not exit (valve opens, no exit). Color coding of the data points corresponds to the transit phenotypes in B. For transit phenotype analysis, the total number of oocytes that exited the spermatheca successfully was compared to the sum of all other phenotypes. Fisher’s exact *t*-tests were used for both population assays and transit phenotype analysis. (C) Exit time of movies in B were compared using One-way ANOVA with a multiple comparison Tukey’s test. Representative normalized Ca^2+^ traces and kymograms of movies in B (D-I) are shown with time of entry, distal neck closure, and time the sp-ut valve opens and closes indicated. Levels of Ca^2+^ signal were normalized to 30 frames before oocyte entry. Kymograms generated by averaging over the columns of each movie frame (see methods). Refer to S3A, S3E, S3F Fig and S5A, S4C, S4D Figs for additional Ca^2+^ traces, and S6D and S6E Fig and S9B and S9C Fig for additional kymograms. (J-M) Color coding of the data points corresponds to the transit phenotypes in A. (J) The number of Ca^2+^ peaks and (K) the peaks per second was determined for wild type animals treated with control RNAi, *gpb-1(RNAi)* and *gpb-2(RNAi)*, and GSA-1(GF) animals treated with control RNAi, *gpb-1(RNAi)*, and *gpb-2(RNAi)*. The amount of time after oocyte entry required to reach either the (L) half maximum or (M) maximum Ca^2+^ signal was quantified for wild type animals treated with control RNAi, GSA-1(GF) animals treated with control RNAi, *gpb-1(RNAi)*, and *gpb-2(RNAi)*. These values were compared using one-way ANOVA with a multiple comparison Tukey’s test. and compared using Fishers exact *t*-test. Stars designate statistical significance (**** p<0.0001, *** p<0.005, ** p<0.01, * p<0.05).

Heterotrimeric G proteins consist of an α, a β and a γ subunit. The β and γ subunits are closely associated and can be considered one functional unit. Activation of the Gα subunit causes Gα to dissociate from Gβγ, and both can then initiate downstream signaling pathways [56]. Therefore, we next explored potential roles for βγ in the spermatheca. Depletion of GPB-1/β resulted in a significant increase in the percent of occupied spermathecae (34%, n = 99) compared to control RNAi treated animals (18% occupied, n = 300; Fig 6A). GPB-1 depletion also resulted in a significant number of spermathecae occupied by a small piece of an embryo (Fig 6A). Surprisingly, while GPB-2 depletion showed no significant trapping in freely moving animals (Fig 6A), when the animals were immobilized for imaging, embryos failed to exit the spermatheca in 60% of *gpb-2(RNAi)* movies (n = 10) (Fig 6B), suggesting animals may be able to compensate somewhat for the loss of GPB-2 with body wall muscle contraction. Only 69% of *gpb-1(RNAi)* ovulations trapped, (Fig 6B). Of the ovulations that were able to exit successfully, neither *gpb-1(RNAi)* nor *gpb-2(RNAi*) on their own affected the time required for the embryo to exit the spermatheca (Fig 6C). Depletion of neither GPC-1/γ nor GPC-2/γ significantly affected ovulation when depleted via RNAi (Fig 6A). Similarly, neither the *gpc-1(pk298)* null allele, nor treating *gpc-1(pk298)* with *gpc-2* RNAi resulted in ovulation defects. These data suggest GPB-1 may be associated with GSA-1 in the spermatheca (Fig 6A), but identification of the relevant γ subunits requires further investigation. Insufficient knockdown of GPC-1/γ nor GPC-2/γ may explain the lack of phenotype.

In order to explore a role for GPB-1 and GPB-2 in Ca^2+^ signaling, we observed ovulations in GCaMP3 expressing animals treated with *gpb-1(RNAi)* and *gpb-2(RNAi)*. Compared to wild type movies, in which oocyte entry triggers a pulse of Ca^2+^ in the sp-ut valve, followed by Ca^2+^ oscillations in the spermathecal bag and sp-ut valve that increase in intensity until the embryo exits (Fig 6D), both *gpb-1(RNAi)* and *gpb-2(RNAi)* resulted in elevated and/or prolonged Ca^2+^ signal in the sp-ut valve and very little Ca^2+^ signal in the spermathecal bag (Fig 6E, Fig 6F, S2E Fig). While loss of GPB-1 or GPB-2 did not significantly reduce the overall number of peaks (Fig 6J), the longer duration of the transits resulted in reduced peak frequencies (Fig 6K). Because little rise in Ca^2+^ is observed, the time to half max (Fig 6L) and time to max (Fig 6M) are short for both *gpb-1* and *gpb-2(RNAi)*. Loss of the β subunits produces similar phenotypes to those observed for *gsa-1(RNAi)*, suggesting GPB-1 and GPB-2 are required for the activation of Ca^2+^ signaling by GSA-1.

Activation of Gα liberates the Gβγ subunits, which can independently initiate signaling pathways [56]. In order to determine if activated signaling by Gβγ could partially explain the GSA-1(GF) phenotypes, we depleted GPB-1 and GPB-2 in the GSA-1(GF) background. First, we assessed the effect on GSA-1(GF) transit phenotypes. In GSA-1(GF) animals, even though the sp-ut valve opens, ~30% of embryos remained inside the spermatheca (n = 19; Fig 6B). Depletion of GPB-1 on its own resulted in significant trapping, with most spermathecae occupied with an embryo (36%) or an embryo fragment (26%) (n = 99; see Fig 6A). When GPB-1 was depleted in the GSA-1(GF) background, no successful transits were observed, with 40% of ovulations resulting in trapping, and 60% of ovulations in which the valve opened but the embryo was unable to exit (n = 5; Fig 6B). In contrast, in GSA-1(GF) *gpb-2(RNAi)* animals, the trapping phenotype did not differ significantly from GSA-1(GF) alone (Fig 6B). However, the significantly longer exit time observed in GSA-1(GF) animals was suppressed by *gpb-2(RNAi)* to wild type levels (Fig 6C).

As previously described, GSA-1(GF) results in strong Ca^2+^ pulses that propagate across the tissue (Fig 6G). When GPB-1 was depleted in GSA-1(GF) expressing animals, other than a brief pulse upon entry, only low amplitude peaks were observed in either the sp-ut valve or in the spermatheca bag (Fig 6H and S2E Fig). This result suggests GSA-1 requires GPB-1 to stimulate Ca^2+^ release, and is consistent with the model that binding to the Gβγ subunit GPB-1 is required for activation of GSA-1. In contrast, depletion of GPB-2 in GSA-1(GF) animals resulted in Ca^2+^ dynamics more similar to those observed in wild type animals (Fig 6I). Depletion of GPB-1 or GPB-2 did not reduce the number of peaks (Fig 6J) or peak frequency (Fig 6K) in GSA-1(GF) animals, although the peaks in the GSA-1(GF); *gpb-1(RNAi)* animals are much lower amplitude than in GSA-1(GF). This feature results in minimal increase in Ca^2+^ levels, reflected in the time to half max (Fig 6L) and time to max (Fig 6M), which were reached shortly after entry in GSA-1(GF) animals treated with *gpb-1(RNAi)*. In contrast, when GSA-1(GF) animals were treated with *gpb-2(RNAi)*, the time to max Ca^2+^ signal was reduced to wild type levels (Fig 6M). These results, in combination with the rescued exit times (Fig 6C), suggest loss of GPB-2 might suppress GSA-1(GF) activity to more wild type levels. Alternatively, the Gβs could be activating parallel signaling pathways needed for Ca^2+^ release, potentially including other Gα subunits active in the spermatheca.

## Discussion

Ca^2+^ and cAMP are important second messengers with key roles in a diverse set of biological processes. Here, we show that GSA-1/Gα_s_ and KIN-1/PKA-C function to regulate Ca^2+^ release and coordinated contractility in the *C*. *elegans* spermatheca. The G_β_ subunits GPB-1 and GPB-2 are required for activation of GSA-1/Gα_s_, and phosphodiesterase PDE-6 functions in the spermatheca to regulate Ca^2+^ levels and overall tissue contractility.

**Fig 7:**
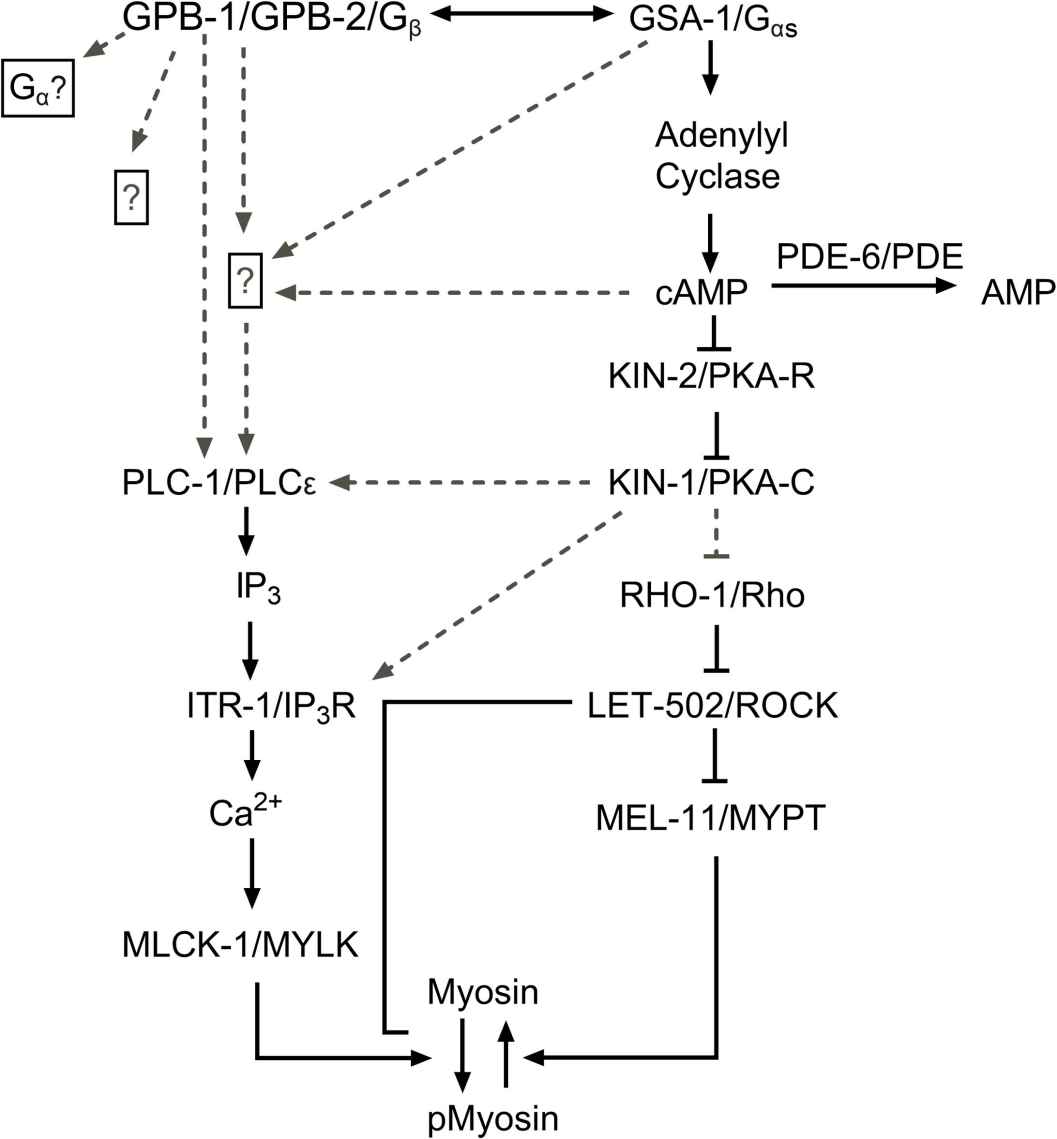
A working model of the spermathecal Ca^2+^ signaling network. We propose KIN-1 and KIN-2 are working downstream of GSA-1, while GPB-1 and GPB-2 are either working together with or in parallel to GSA-1 to regulate Ca^2+^ signaling and spermathecal contractility. Dashed lines indicate possible interactions.

In the spermatheca, oocyte entry triggers Ca^2+^ waves, which propagate across the spermatheca and trigger spermathecal contractility. We found that loss of either GSA-1/Gα_s_ or KIN-1/PKA-C leads to low levels of Ca^2+^ signal in the spermathecal bag, while increasing GSA-1 activity with a gain of function allele or activating PKA through loss of the regulatory subunit, KIN-2/PKA-R, leads to strong Ca^2+^ pulses, even in the absence of oocyte entry. This suggests GSA-1/Gα_s_ and KIN-1/PKA-C stimulate Ca^2+^ release in the spermatheca. Previous work in our lab has shown that PLC-1/phospholipase C-ε is necessary to stimulate Ca^2+^ release in the spermatheca [5]. Here, we show that loss of PLC-1 can block the Ca^2+^ release observed in GSA-1(GF) and *kin-2(RNAi)* animals, suggesting PLC-1 acts either downstream or in parallel with Gα_s_ and PKA (Fig 7). Mechanisms by which PKA could stimulate Ca^2+^ release include activation of the ITR-1/IP_3_ receptor, or activation of plasma membrane channels such as stretch-sensitive TRPV channels [57]. For example, in mouse cardiomyocytes, Gα activation can stimulate Ca^2+^ release through exchange protein directly activated by cAMP (EPAC) and Rap1 [58,59].

Heterotrimeric G-proteins consist of an α and a βγ subunit, which, when activated by an upstream G-protein coupled receptor (GPCR) or G-protein regulator (GPR), dissociate and independently activate signaling cascades [60–62]. *C*. *elegans* expresses numerous GPCRs [63], which has hampered our efforts to identify the upstream activator of Gα. A set of GPCRs expressed in the spermatheca has recently been published [64], which may facilitate the identification of a receptor that acts upstream of GSA-1. Alternatively, GPRs can facilitate the activation of G proteins via GPCR-independent mechanisms [20,28]. Regardless of the trigger, we show both Gα and Gβ signaling are important for regulated Ca^2+^ release in the spermatheca. The presence of Gβ subunit GPB-1 is required for the activation of the heterotrimer, while GPB-2 may play a more modulatory role, however we cannot rule out that the observed differences between *gpb-1(RNAi)* and *gbp-2 (RNAi)* may be due to differences in RNAi efficiency. Not only are the Gβs critical for Gα activation, the Gβγ subunits may also be activating other downstream effectors critical for Ca^2+^ release. For example, in COS-7 cells, PLC-ε can be activated by Gβγ subunits [65], which offers a promising alternative. Future work is needed to explore a possible connection between Gβγ and PLC-1 in the spermatheca.

Contraction of the spermatheca requires both Ca^2+^ and Rho signaling. Therefore, the observed transit defects could be due to GSA-1/Gα_s_ and KIN-1/PKA-C regulation of either, or both, of these signaling pathways. Because *kin-2(RNAi)*, which activates KIN-1/PKA-R and stimulates Ca^2+^ release, is unable to produce contractions sufficient to expel the embryo from the spermatheca, this implies PKA may also regulate the Rho side of the pathway. In SH-EP cells, PKA has been shown to phosphorylate and inhibit Rho through stabilization of the inactive RhoGDI-bound state [44,66] Given that the Rho phosphorylation site is conserved in the *C*. *elegans* RHO-1, it is possible that knockdown of KIN-2 results in an increased inhibition of RHO-1 thus resulting in decreased tissue contractility in *kin-2(RNAi)* animals.

The sp-ut valve is a syncytium of 4 cells that prevents premature release of oocytes into the uterus upon entry in the spermatheca. This allows for enough time for the oocyte to become fertilized and form an eggshell, after which the sp-ut dilates and allows passage of the embryo into the uterus [67]. Here, we show that PKA regulates Ca^2+^ release and contractility of the sp-ut valve. Depletion of KIN-1/PKA-C activity results in increased Ca^2+^ and a valve that remains closed. In contrast, hyperactive PKA-C activity (through *kin-2(RNAi)*) results in an sp-ut valve that opens prematurely, perhaps accounting for the short dwell times observed when PKA-R is depleted (see Fig 1E). Decreasing signal through PKA/KIN-1 or Gα_s_ /GSA-1 knockdown results in a surplus of Ca^2+^ in the sp-ut valve, whereas increasing signaling through GSA-1(GF) results in an extended quiet Ca^2+^ period in the sp-ut valve. This suggests PKA/KIN-1 and Gα_s_ /GSA-1 are required for Ca^2+^ inhibition in the sp-ut valve even though they are required for Ca^2+^ release in the spermathecal bag. Little is known about the signaling networks that regulate contractility in the sp-ut valve. PLC-1 is not expressed in the sp-ut valve [3], necessitating a different mechanism of Ca^2+^ regulation from the spermathecal bag. Phosphorylation of IP_3_Rs by PKA-C has been shown to decrease Ca^2+^ release in rat brain cells [68], which could help to explain the Ca^2+^ excess seen in the sp-ut valve. Other mechanisms by which PKA has been described to lower Ca^2+^ include increasing the activity of SERCA pumps, which pump Ca^2+^ back into the ER, by phosphorylating and dissociating phospholamban [69,70]. However, there is no obvious phospholamban homolog in *C*. *elegans*. PKA can inhibit PLC-β [71], which would result in decreased Ca^2+^ release. However, EGL-8/PLC- β is not expressed in either the spermatheca or sp-ut valve [72]. Future study may reveal the details of how PKA regulates Ca^2+^ signaling in the sp-ut valve.

Regulating the correct force, timing, and direction of contraction in biological tubes is crucial for living organisms. Dysfunction in the regulatory networks controlling these dynamics leads to diseases such as heart disease and asthma [42,73]. The signaling networks that control Ca^2+^ oscillations and actomyosin contractility in smooth muscle in humans are conserved in the *C*. *elegans* spermatheca. In this study, we identified Gα_s_ and PKA as key players in the spermatheca, where Gα_s_ acts upstream of PKA to regulate the spatiotemporal regulation of Ca^2+^ release and contractility in the spermatheca. Because PKA is implicated in a variety of biological processes, understanding its control and targets may give us insights into the diseases that occur when this control goes awry.

## Materials and methods

### Strains and culture

Nematodes were grown on nematode growth media (NGM) (0.107 M NaCl, 0.25% wt/vol Peptone (Fischer Science Education), 1.7% wt/vol BD Bacto-Agar (FisherScientific), 0.5% Nystatin (Sigma), 0.1 mM CaCl_2_, 0.1 mM MgSO_4_, 0.5% wt/vol cholesterol, 2.5 mM KPO_4_) and seeded with *E*. *coli OP50* using standard *C*. *elegans* techniques [74]. Nematodes were cultured at 23°C unless specified otherwise. All Ca^2+^ imaging was acquired using the strain UN1108 *xbls1101 [fln-1p*::*GCaMP3*,*rol-6]* except for experiments utilizing the GSA-1 gain of function mutant KG524 *GSA-1(GF)*. These animals were crossed into UN1417, a separate integration event of *xbIs1108 [fln-1p*::*GCaMP3]*.

### Construction of the transcriptional and translational reporter of GSA-1

The *gsa-1* promoter (1.6 kb upstream of GSA-1 start codon) was amplified from *C*. *elegans* genomic DNA using primers with PstI and BamHI 5’ extensions and ligated into pPD95_77 (Fire Lab) upstream of GFP creating pUN783. To make a translational fusion *gsa-1* was amplified without its stop codon from *C*. *elegans* coding DNA using primers with BamHI 5’ extensions and ligated between the *gsa-1* promoter and GFP of pUN783 creating pUN810. Transgenic animals were created by microinjecting a DNA solution of 20 ng/μl of pUN783 or pUN810 and 50 ng/μl of pRF4 *rol-6* (injection marker) into N2 animals. Roller animals expressing GFP were segregated to create the transgenic lines UN1727 (transcriptional reporter) and UN1742 (translational reporter) respectively.

### RNA interference

The RNAi protocol was performed essentially as described in Timmons et al (1998) [75]. HT115(DE3) bacteria (RNAi bacteria) transformed with a dsRNA construct of interest was grown overnight in Luria Broth (LB) supplemented with 40 μg/ml ampicillin and seeded (150 μl) on NGM plates supplemented with 25 μg/ml carbenicillin and disopropylthio-β-galactoside (IPTG). Seeded plates were left for 24–72 hours at room temperature (RT) to induce dsRNA expression. Empty pPD129.36 vector (“Control RNAi”) was used as a negative control in all RNAi experiments.

Embryos from gravid adults were collected using an alkaline hypochlorite solution as described by Hope (1999) and washed three times in M9 buffer (22 mM KH_2_PO_4_, 42 mM NaHPO_4_, 86 mM NaCl, and 1 mM MgSO_4_) (‘egg prep’). Clean embryos were transferred to supplemented NGM plates seeded with HT115(DE3) bacteria expressing dsRNA of interest and left to incubate at 23°C for 50–56 hours depending on the experiment. In experiments where larvae, rather than embryos, were transferred to RNAi plates (*kin-1(RNAi)* and *kin-2(RNAi))*, adults were ‘egg prepped’ into Control RNAi and left to incubate at 23°C for 32–34 hours, after which they were moved to bacterial lawns expressing the dsRNA of interest and returned to 23°C, and imaged 50–56 after the egg prep.

### Population assay

Embryos collected via an ‘egg prep’ as previously described were plated on supplemented NGM seeded with RNAi bacterial clones of interest. Plates were incubated at 23°C for 54–56 hours or until animals reached adulthood. Upon adulthood nematodes were killed in a drop of 0.08 M sodium azide (NaAz) and mounted on 2% agarose pads to be visualized using a 60x oil-immersion objective with a Nikon Eclipse 80i epifluorescence microscope equipped with a Spot RT3 CCD camera (Diagnostic instruments; Sterling Heights, MI, USA). Animals were scored for the presence or absence of an embryo in the spermatheca as well as entry defects such as gonad arms containing endomitotically duplicating oocytes (Emo) [51]. A Fisher’s exact *t*-test (two dimensional *x*^*2*^ analysis) using GraphPad Prism statistical software was used to compare the percent of occupied spermathecae between control RNAi and all other RNAi treatments.

### Wide-field Fluorescence microscopy image acquisition and processing

All Differential Interference Contrast (DIC) and fluorescent images were taken using a 60x oil-immersion objective with Nikon Eclipse fluorescent microscope equipped with a Spot RT3 CCD camera (Diagnostic instruments; Sterling Heights, MI, USA) or Spot RT39M5 with a 0.55x adapter unless otherwise stated. Fluorescence excitation was provided by a Nikon Intensilight C-HGFI 130W mercury lamp and shuttered with a SmartShutter (Sutter Instruments, Novato CA, USA). For acquisition of time-lapse images young adult animals were immobilized with 0.05 micron polystene polybeads diluted 1:2 in water (Polysciences Inc., Warrington, PA, USA) and mounted on slides with 5% agarose pads. Time lapse GCaMP imaging was captured at 1 frame per second, with an exposure time of 75 ms and a gain of 8 for movies obtained with the Spot RT3CCD camera, and with an exposure time of 20 ms and a gain of 8 for movies obtained with the Spot RT39M5. Time-lapse images were only taken of the first 3 ovulations, with preference for the 1^st^ ovulation. The same microscopy image capture parameters were maintained for all imaging.

All time-lapse GCaMP3 images were acquired as 1600x1200 pixels for the Spot RT3 OCCD camera or 2448x2048 for the RT39M5 camera and saved as 8-bit tagged image and file format (TIFF) files. All image processing was done using a macro on Image J [76]. All time-lapse images were oriented with the sp-ut valve on the right of the frame and registered to minimize any body movement of the paralyzed animal. An 800x400 region of interest for the Spot RT3 OCCD and 942x471 for the RT39M5 camera encompassing the entire spermatheca was utilized to measure the GCaMP3 signal. The average pixel intensity of each frame was calculated using a custom ImageJ macro [5]. Ca^2+^ pixel intensity (F) was normalized to the average pixel intensity of the first 30 frames prior to the start of ovulation (F_0_) and plotted against time. Data analysis and graphing were performed using Matlab and GraphPad Prism. Matlab was used to identify peaks in the GCaMP time series. Specifically, data was smoothed using a moving average of 5 data points via the Matlab ‘smoothdata’ command. Local maxima in the time series with a minimum prominence of 0.1 units and a minimum width of 5 units were then identified using the Matlab ‘findpeaks’ command. These standardized settings were used to analyze all Ca^2+^ traces. Microsoft Excel was used to quantify the amount of time after oocyte entry required to reach either the half the maximum or maximum Ca^2+^ signal as identified by ‘findpeaks’. To determine the rate at which the Ca^2+^ signal changed with time, Microsoft Excel was used to calculate the variance of the first derivative for each time series. Kymograms were generated using an ImageJ macro that calculated the average pixel intensity of each column of a frame and condensing it down to one line per frame of the time-lapse image. Every frame of the time-lapse image was stacked to visualize Ca^2+^ dynamics of representative ovulations in both space and time. The Fire look up table color scale was applied to the kymograms using Image J. All Matlab and ImageJ scripts are available upon request.

### Time point metrics

During time-lapse image processing four timepoints are recorded for each ovulation. These include (1) start of oocyte entry: the time when the spermatheca is beginning to be pulled over the incoming oocyte, (2) distal spermathecal closure: the time when the distal spermatheca closes over the oocyte and the oocyte is completely in the spermatheca, (3) sp-ut valve opening: the time when sp-ut valve starts to open and spermathecal exit begins (4) sp-ut valve closure: the time when the sp-ut valve completely closes and the embryo is fully in the uterus. These times were used to calculate both the total transit time, dwell time and exit time of each ovulation. The total transit time is defined as the time from the start of oocyte entry to sp-ut valve closing. Dwell time is defined as the amount of time between the distal neck closing to the sp-ut valve opening. Dwell time captures how long the embryo is in the spermatheca with both the neck and sp-ut valve closed. Exit time is defined as the amount of time from the sp-ut valve opening until the embryo is completely in the uterus and the sp-ut valve closes again behind it.

### Statistics

Either a Fishers exact *t*-test (two dimensional *x*^*2*^ analysis) or a one-way ANOVA with a multiple comparison Tukey’s test were conducted using GraphPad Prism on total, dwell and exit transit times of ovulations acquired via time-lapse imaging. For population assays, statistics were performed using the total number of unoccupied spermathecae compared with the sum all other phenotypes. N is the total number of spermathecae counted. For transit phenotype analysis, statistics were performed using the total number of oocytes that exited the spermatheca successfully compared to the sum of all other phenotypes. Fisher’s exact *t*-test were used for both population assays and transit phenotype analysis. Stars designate statistical significance (**** p<0.0001, *** p<0.005, ** p<0.01, * p<0.05).

## Supporting information

S1 Movie Ca^2+^ oscillations observed in the spermatheca of a wild type hermaphrodite grown on control RNAi. A representative wild type ovulation of an animal expressing GCaMP3. Oocyte enters from the right at 30 seconds. There is an initial pulse of Ca^2+^ in the sp-ut valve, followed by Ca^2+^ oscillations that originate from the distal spermatheca on the right, and propagate to the proximal spermatheca on the left until embryo exit.
(AVI)

S2 Movie Ca^2+^ oscillations observed in the spermatheca of a wild type hermaphrodite grown on *gsa-1(RNAi)*. A representative *gsa-1(RNAi)* ovulation. Oocyte enters from the right at 30 seconds. The sp-ut valve shows increased Ca^2+^ signal after oocyte entry, and signal remains elevated throughout movie. Embryo is trapped in the spermatheca.
(AVI)

S3 Movie Ca^2+^ oscillations observed in the spermatheca of a wild type hermaphrodite grown on *kin-1(RNAi)*. A representative *kin-1(RNAi)* ovulation. Oocyte enters from the right at 30 seconds. The sp-ut valve shows increased Ca^2+^ signal after oocyte entry, and signal remains elevated throughout movie. Additionally, there is increased Ca^2+^ signal only in the proximal spermatheca. Embryo is pushed back into the gonad arm and then re-enters the spermatheca.
(MOV)

S4 Movie Ca^2+^ oscillations observed in the spermatheca of a wild type hermaphrodite grown on *kin-2(RNAi)*. A representative *kin-2(RNAi)* ovulation. Oocyte enters from the right at 30 seconds. Pulses of Ca^2+^ propagate from distal to proximal immediately after oocyte entry. The sp-ut valve opens almost immediately. The embryo did not exit during the duration of the movie.
(MOV)

S5 Movie Ca^2+^ oscillations observed in an empty spermatheca of a GSA-1(GF) animal. Pulses of Ca^2+^ propagate from distal to proximal immediately after oocyte entry. The sp-ut valve opens almost immediately. The embryo did not exit during the duration of the movie.
(AVI)

S1 Fig Frames from a representative video of a *kin-2(RNAi)* transit. (A) Frames taken from S4 Movie showing Ca^2+^ signaling in a *kin-2(RNAi)* transit. Ca^2+^ repeatedly increases, peaks, and then drops to baseline levels. These pulses travel from the distal spermatheca through the bag to the proximal sp-ut valve.
(TIF)

S2 Fig Ca^2+^ traces differ in degree of variability. As a measure of how the traces change with time, the variance of the first derivative is compared between (A) wild type animals treated with control RNAi, *gsa-1(RNAi)*, *kin-1(RNAi)*, and *kin-2(RNAi)*, (B) wild type animals treated with control RNAi, and GSA-1(GF) animals treated with control RNAi and *kin-1(RNAi)*, (C) GSA-1(GF) animals treated with control RNAi and *plc-1(RNAi)*, and *plc-1* null animals treated with control RNAi, *kin-1(RNAi)*, *and kin-2(RNAi)*, (D) wild type animals treated with control RNAi and *pde-6(RNAi)*, and (E) wild type animals treated with control RNAi, *gpb-1(RNAi)* and *gpb-2(RNAi)*, and GSA-1(GF) animals treated with control RNAi, *gpb-1(RNAi)*, and *gpb-2(RNAi)*, and compared using One-way ANOVA with a multiple comparison Tukey’s test. Stars designate statistical significance (**** p<0.0001, *** p<0.005, ** p<0.01, * p<0.05).
(TIF)

S3 Fig Compilation of all Ca^2+^ traces collected in wild type; *gsa-1*, *kin-1*, *kin-2*, *gpb-1*, *gpb-2*, *pde-6*, and *plc-1* RNAi treated animals. All Ca^2+^ traces of wild type animals treated with (A) control, (B) *gsa-1(RNAi)*, (C) *kin-1(RNAi)*, (D) *kin-2(RNAi)*, (E) *gpb-1(RNAi)*, (F) *gpb-2(RNAi)*, (G) *pde-6(RNAi)*, and (H) *plc-1(RNAi)*. Pixel intensity (F) was normalized to the average pixel intensity of the first 30 frames prior to the start of ovulation (F_0_) and plotted against time. Ovulations that exit successfully, trap, return to gonad arm, and ovulations in which the valve opens but the embryo does not exit are annotated.
(TIF)

S4 Fig Compilation of all kymograms collected in wild type; *gsa-1*, *kin-1* and *kin-2* RNAi treated animals. Kymograms of wild type animals treated with (A) control RNAi, (B) *gsa-1(RNAi)*, (C) *kin-1(RNAi)*, (D) and *kin-2(RNAi)* ovulation movies. Kymograms were generated by averaging over the columns of each movie frame.
(TIF)

S5 Fig Compilation of all Ca^2+^ traces collected in GSA-1(GF), *kin-1*, *plc-1*, *gpb-1*, and *gpb-2* RNAi treated animals. All Ca^2+^ traces of GSA-1(GF) animals treated with (A) control, (B) *kin-1(RNAi)*, (C) *gpb-1(RNAi)*, (D) *gpb-2(RNAi)*, and (E) *plc-1(RNAi)*. Pixel intensity (F) was normalized to the average pixel intensity of the first 30 frames prior to the start of ovulation (F_0_) and plotted against time. Ovulations that exit successfully, trap, return to gonad arm, and ovulations in which the valve opens but the embryo does not exit are annotated.
(TIF)

S6 Fig Compilation of all kymograms collected in GSA-1(GF); *kin-1*, *plc-1*, *gpb-1*, and *gpb-2* RNAi treated animals. Kymograms of GSA-1(GF) animals treated with (A) control RNAi, (B) *kin-1(RNAi)*, (C) *plc-1(RNAi)*, (D) *gpb-1(RNAi)*, (E) and *gpb-2(RNAi)* ovulation movies. Kymograms were generated by averaging over the columns of each movie frame.
(TIF)

S7 Fig Compilation of all Ca^2+^ traces collected in *plc-1(rx1)*; *kin-1* and *kin-2* RNAi treated animals. All Ca^2+^ traces of *plc-1(rx1)* animals treated with (A) control, (B) *kin-1(RNAi)*, and *kin-2(RNAi)*. Pixel intensity (F) was normalized to the average pixel intensity of the first 30 frames prior to the start of ovulation (F_0_) and plotted against time. Ovulations that exit successfully, trap, return to gonad arm, and ovulations in which the valve opens but the embryo does not exit are annotated.
(TIF)

S8 Fig Compilation of all kymograms collected in *plc-1(rx1)*; *kin-1* and *kin-2* RNAi treated animals. Kymograms of *plc*-*1(rx1)* animals treated with (A) control RNAi, (B) *kin-1(RNAi)*, and (C) *kin-2(RNAi)*. Kymograms were generated by averaging over the columns of each movie frame.
(TIF)

S9 Fig Compilation of all kymograms collected in wild type, *pde-6*, *gpb-1* and *gpb-2* RNAi treated animals. Kymograms of wild type animals treated with (A) *pde-6(RNAi)*, (B) *gpb-1(RNAi)*, (C) *gpb-2(RNAi)* ovulation movies. Kymograms were generated by averaging over the columns of each movie frame.
(TIF)

S10 Fig Similar calcium metrics are observed in *pde-6(RNAi)* and GSA-1(GF) animals. Direct comparison of (A) the number of peaks, (B) the number of peaks per second, (C) time to half maximum, and (D) maximum Ca^2+^ signal between *pde-6(RNAi)* and GSA-1(GF) animals.
(TIF)
