# Supplementary figures and images for: Gα/GSA-1 works upstream of PKA/KIN-1 to regulate calcium signaling and contractility in the Caenorhabditis elegans spermatheca
Source: PLoS Genet. 2020 Aug 10;16(8):e1008644. doi: 10.1371/journal.pgen.1008644 (PMC7444582; doi:10.1371/journal.pgen.1008644)

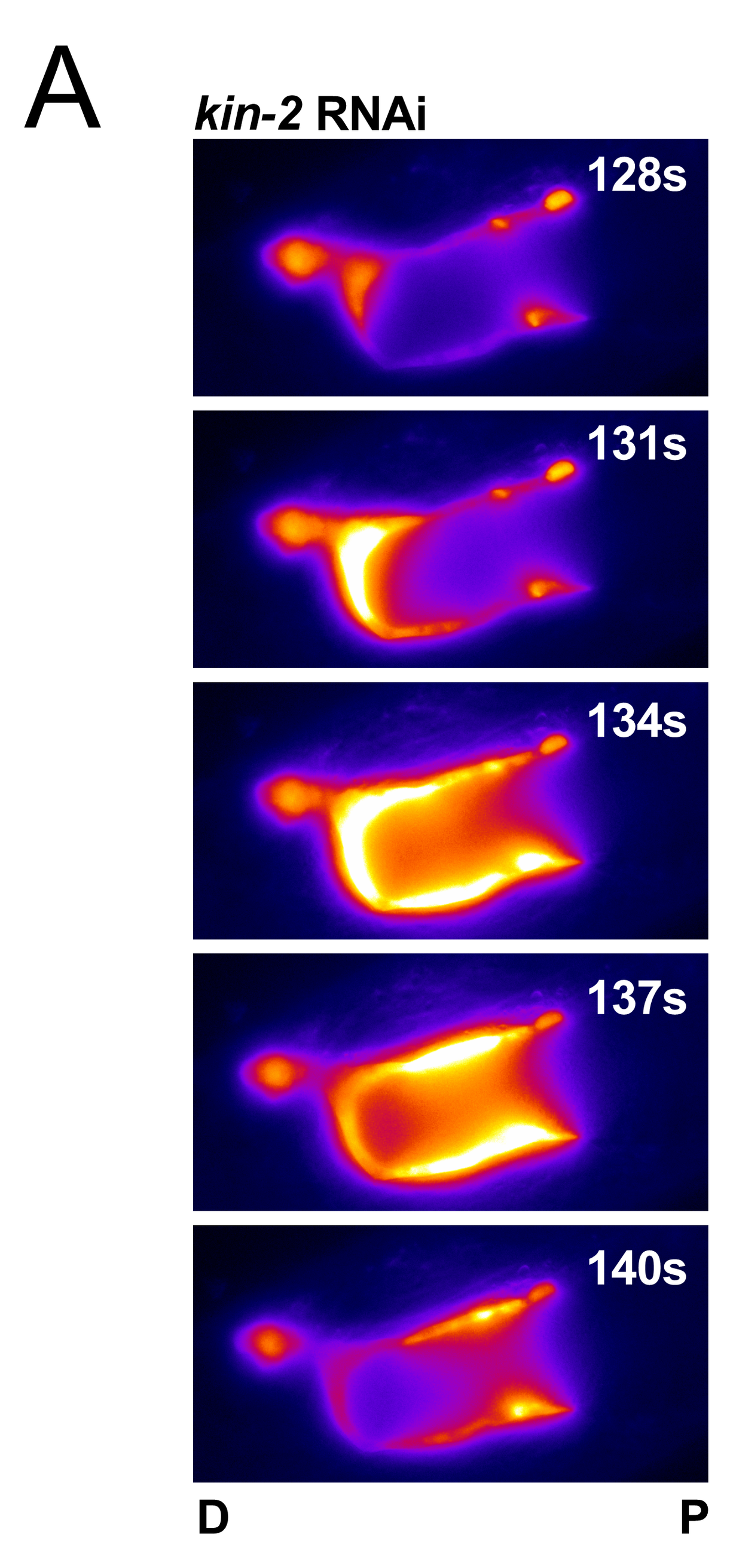

Supplement: S1 Fig — (A) Frames taken from S4 Movie showing Ca2+ signaling in a kin-2(RNAi) transit. Ca2+ repeatedly increases, peaks, and then drops to baseline levels. These pulses travel from the distal spermatheca through the bag to the proximal sp-ut valve. (TIF) [file pgen.1008644.s006.tif]

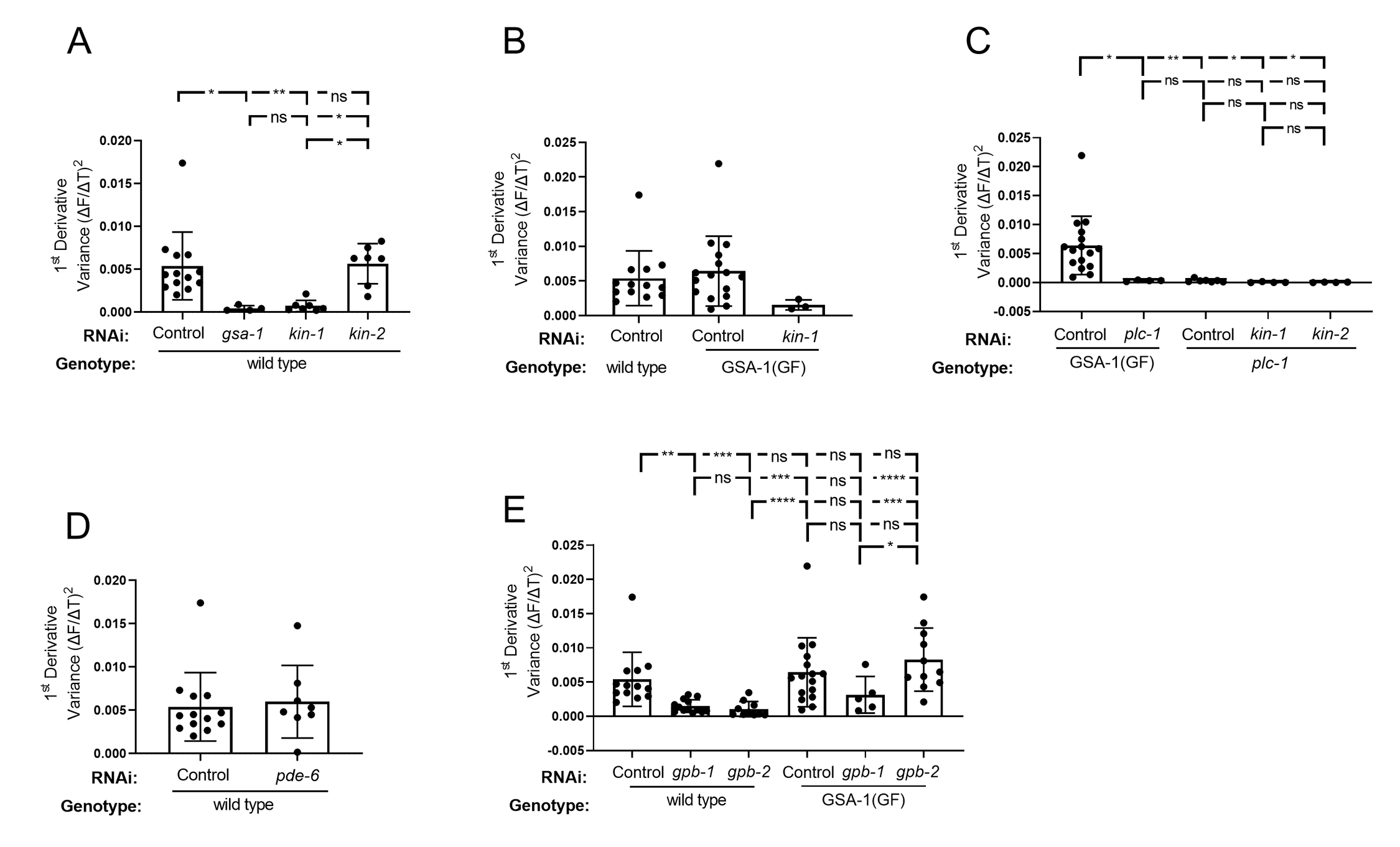

Supplement: S2 Fig — As a measure of how the traces change with time, the variance of the first derivative is compared between (A) wild type animals treated with control RNAi, gsa-1(RNAi), kin-1(RNAi), and kin-2(RNAi), (B) wild type animals treated with control RNAi, and GSA-1(GF) animals treated with control RNAi and kin-1(RNAi), (C) GSA-1(GF) animals treated with control RNAi and plc-1(RNAi), and plc-1 null animals treated with control RNAi, kin-1(RNAi), and kin-2(RNAi), (D) wild type animals treated with control RNAi and pde-6(RNAi), and (E) wild type animals treated with control RNAi, gpb-1(RNAi) and gpb-2(RNAi), and GSA-1(GF) animals treated with control RNAi, gpb-1(RNAi), and gpb-2(RNAi), and compared using One-way ANOVA with a multiple comparison Tukey’s test. Stars designate statistical significance (**** p<0.0001, *** p<0.005, ** p<0.01, * p<0.05). (TIF) [file pgen.1008644.s007.tif]

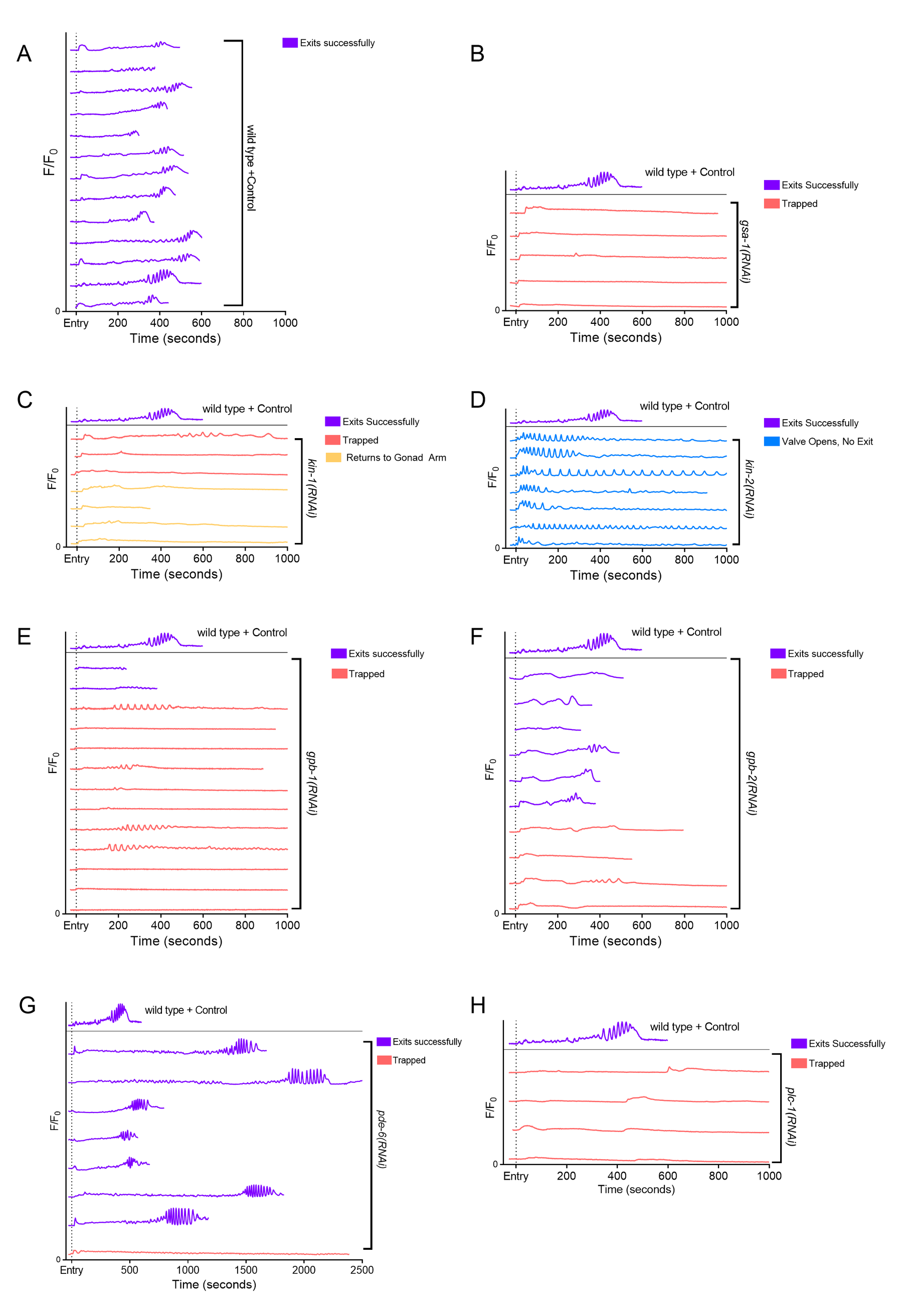

Supplement: S3 Fig — All Ca2+ traces of wild type animals treated with (A) control, (B) gsa-1(RNAi), (C) kin-1(RNAi), (D) kin-2(RNAi), (E) gpb-1(RNAi), (F) gpb-2(RNAi), (G) pde-6(RNAi), and (H) plc-1(RNAi). Pixel intensity (F) was normalized to the average pixel intensity of the first 30 frames prior to the start of ovulation (F0) and plotted against time. Ovulations that exit successfully, trap, return to gonad arm, and ovulations in which the valve opens but the embryo does not exit are annotated. (TIF) [file pgen.1008644.s008.tif]

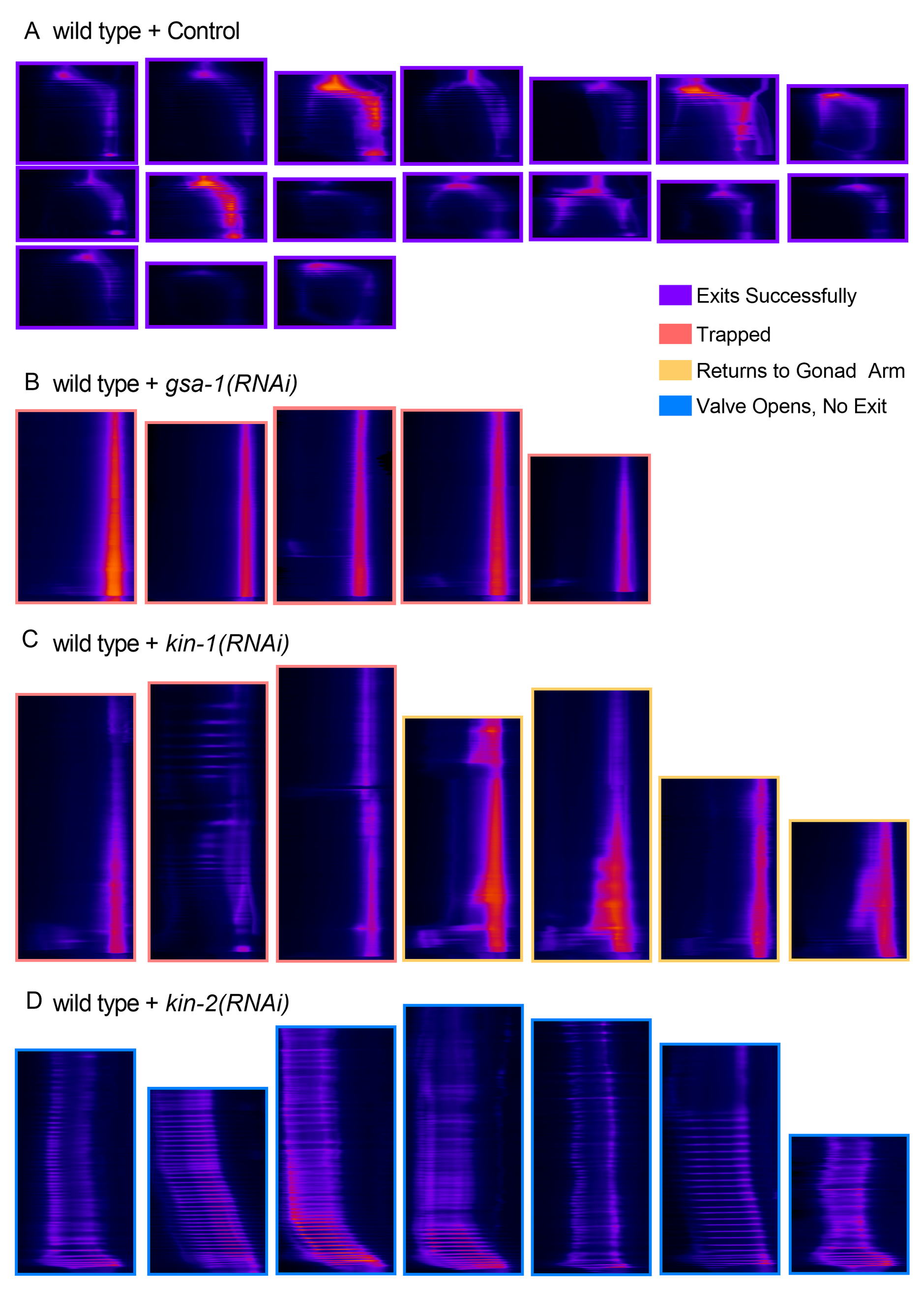

Supplement: S4 Fig — Kymograms of wild type animals treated with (A) control RNAi, (B) gsa-1(RNAi), (C) kin-1(RNAi), (D) and kin-2(RNAi) ovulation movies. Kymograms were generated by averaging over the columns of each movie frame. (TIF) [file pgen.1008644.s009.tif]

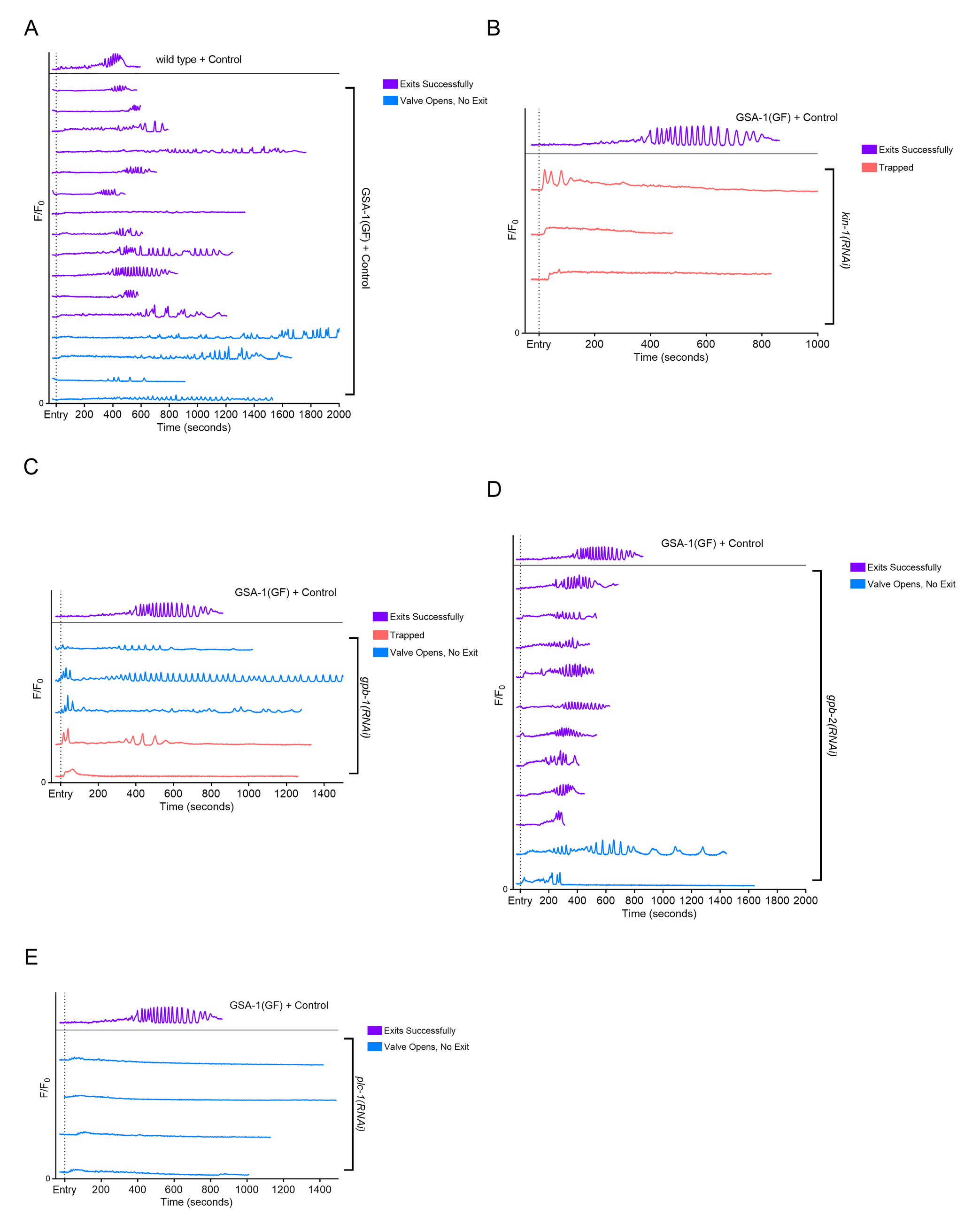

Supplement: S5 Fig — All Ca2+ traces of GSA-1(GF) animals treated with (A) control, (B) kin-1(RNAi), (C) gpb-1(RNAi), (D) gpb-2(RNAi), and (E) plc-1(RNAi). Pixel intensity (F) was normalized to the average pixel intensity of the first 30 frames prior to the start of ovulation (F0) and plotted against time. Ovulations that exit successfully, trap, return to gonad arm, and ovulations in which the valve opens but the embryo does not exit are annotated. (TIF) [file pgen.1008644.s010.tif]

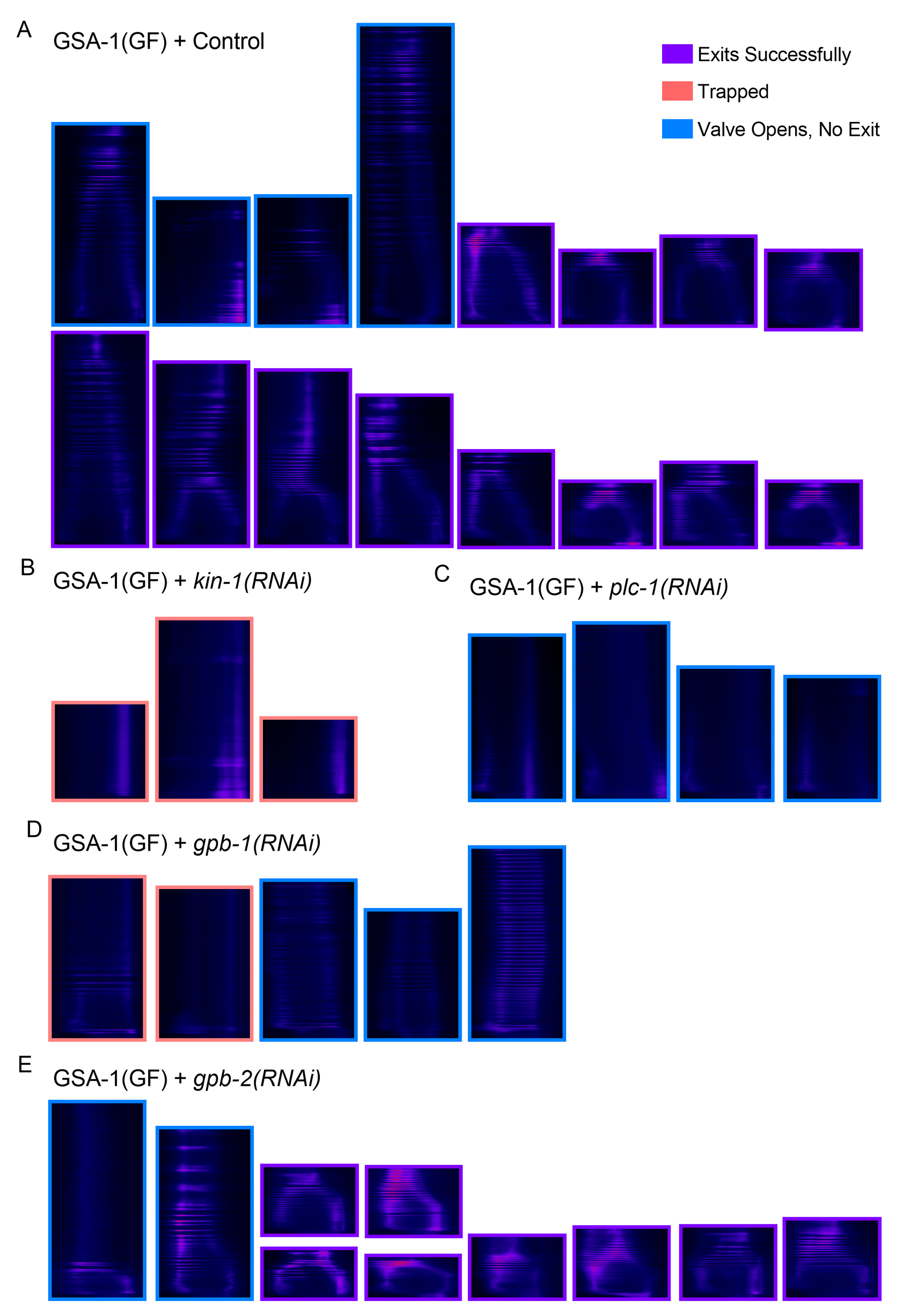

Supplement: S6 Fig — Kymograms of GSA-1(GF) animals treated with (A) control RNAi, (B) kin-1(RNAi), (C) plc-1(RNAi), (D) gpb-1(RNAi), (E) and gpb-2(RNAi) ovulation movies. Kymograms were generated by averaging over the columns of each movie frame. (TIF) [file pgen.1008644.s011.tif]

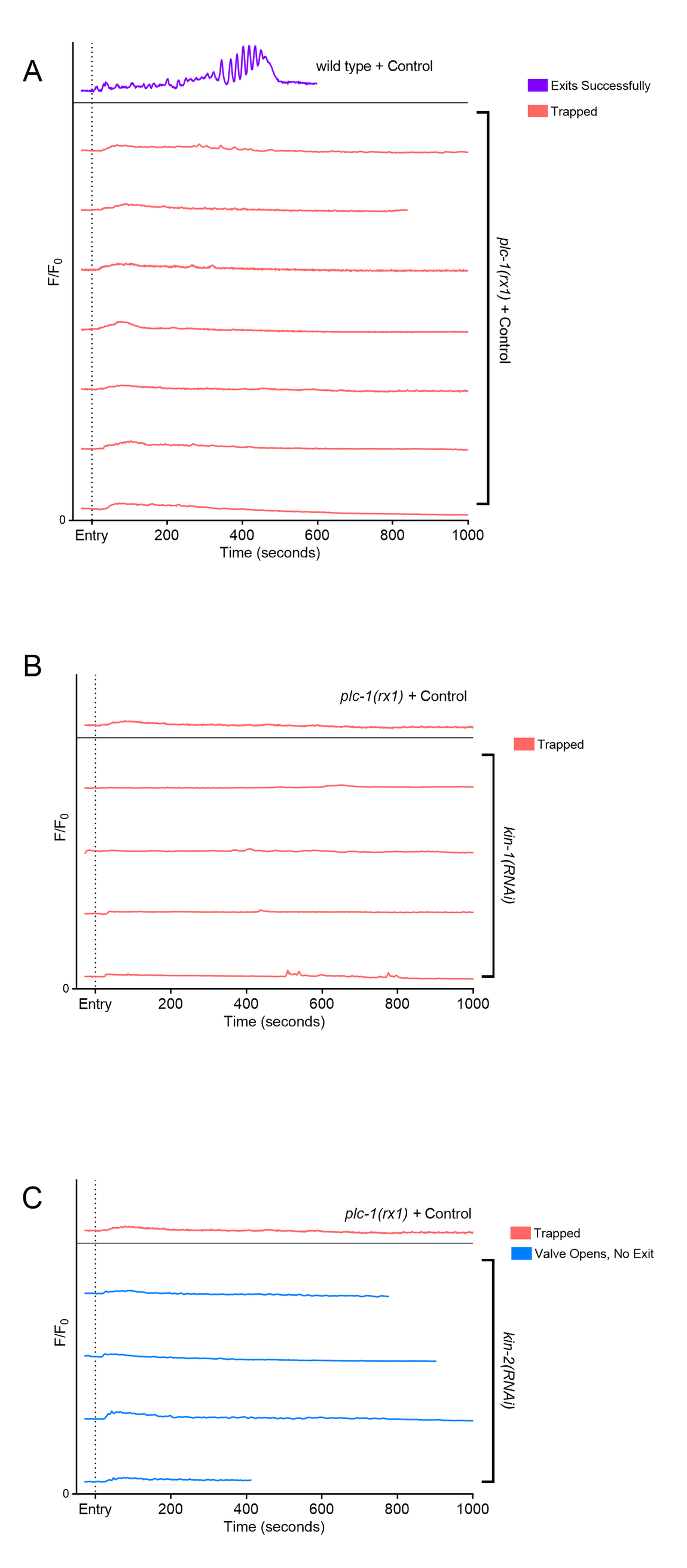

Supplement: S7 Fig — All Ca2+ traces of plc-1(rx1) animals treated with (A) control, (B) kin-1(RNAi), and kin-2(RNAi). Pixel intensity (F) was normalized to the average pixel intensity of the first 30 frames prior to the start of ovulation (F0) and plotted against time. Ovulations that exit successfully, trap, return to gonad arm, and ovulations in which the valve opens but the embryo does not exit are annotated. (TIF) [file pgen.1008644.s012.tif]

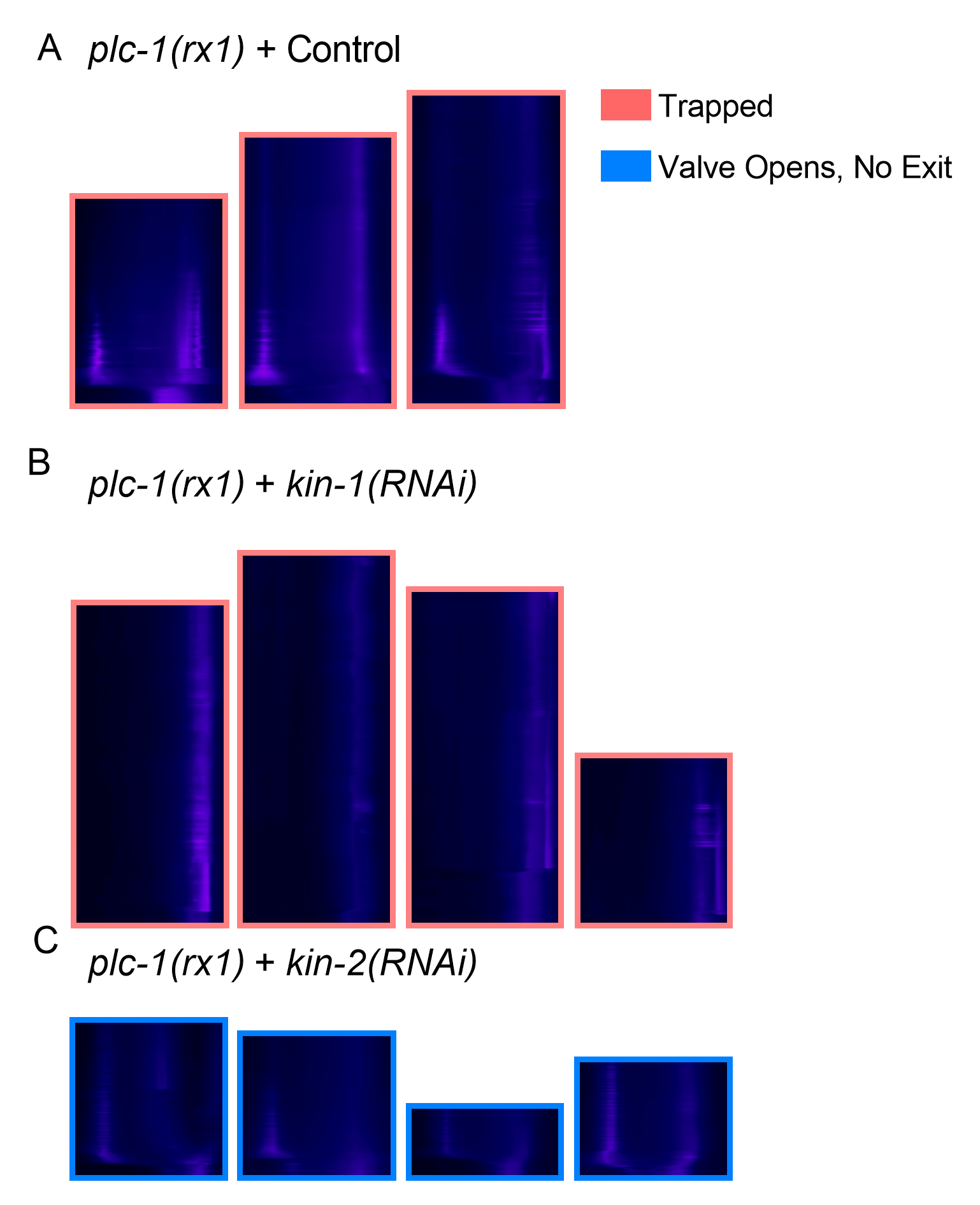

Supplement: S8 Fig — Kymograms of plc-1(rx1) animals treated with (A) control RNAi, (B) kin-1(RNAi), and (C) kin-2(RNAi). Kymograms were generated by averaging over the columns of each movie frame. (TIF) [file pgen.1008644.s013.tif]

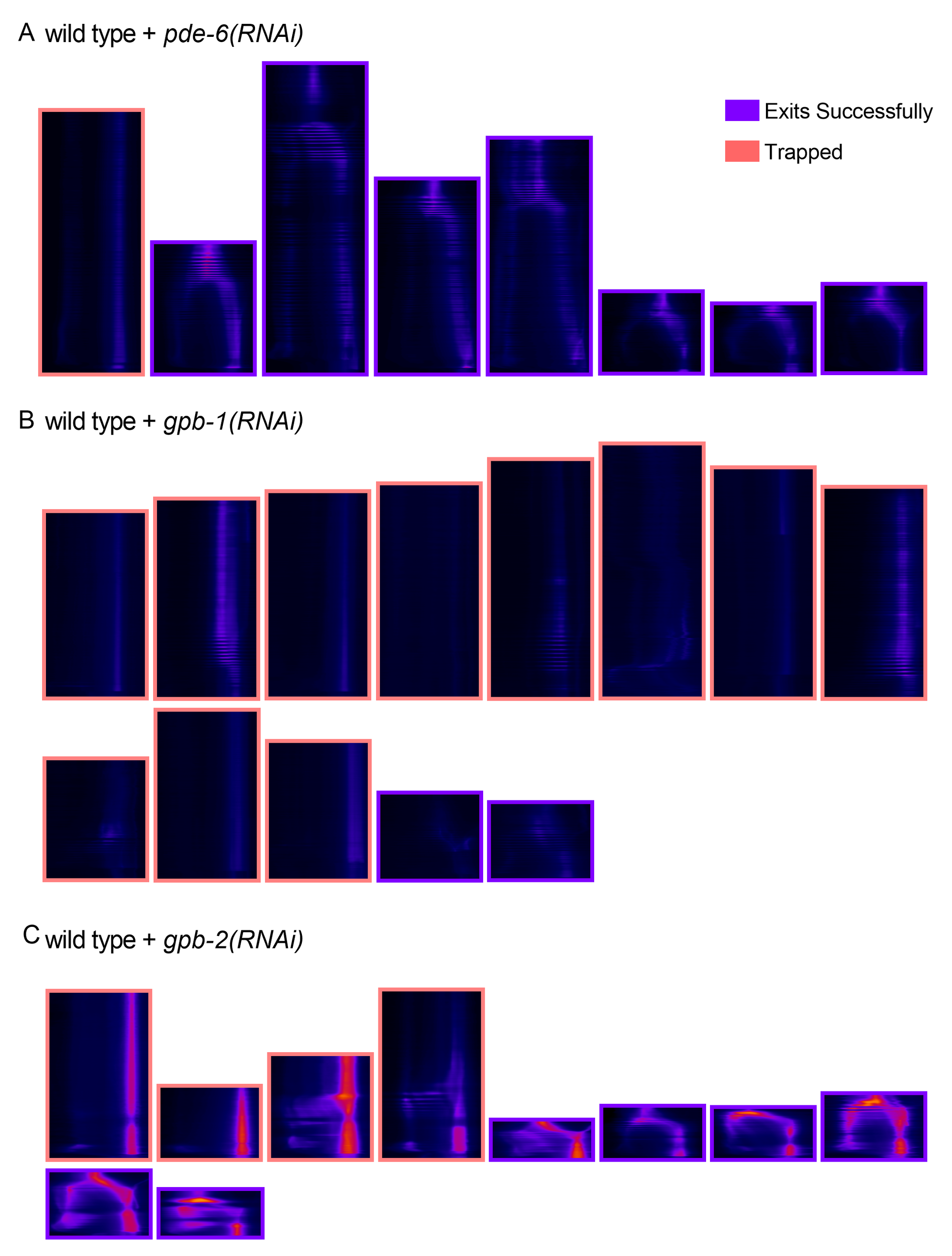

Supplement: S9 Fig — Kymograms of wild type animals treated with (A) pde-6(RNAi), (B) gpb-1(RNAi), (C) gpb-2(RNAi) ovulation movies. Kymograms were generated by averaging over the columns of each movie frame. (TIF) [file pgen.1008644.s014.tif]

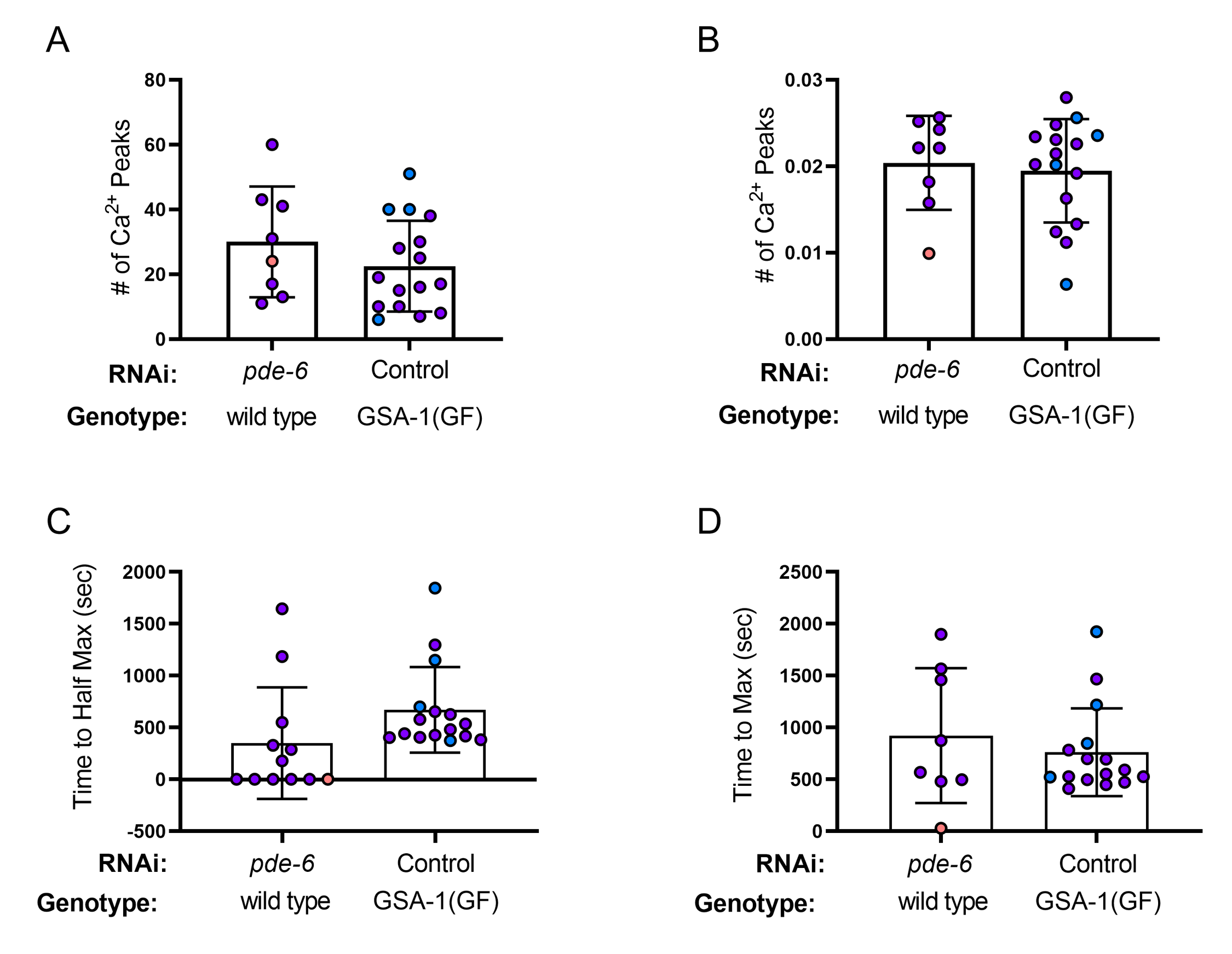

Supplement: S10 Fig — Direct comparison of (A) the number of peaks, (B) the number of peaks per second, (C) time to half maximum, and (D) maximum Ca2+ signal between pde-6(RNAi) and GSA-1(GF) animals. (TIF) [file pgen.1008644.s015.tif]
